# An Analytical Framework for Studying Small-Number Effects in Catalytic Reaction Networks: A Probability Generating Function Approach to Chemical Master Equations

**DOI:** 10.3389/fphys.2016.00089

**Published:** 2016-03-24

**Authors:** Masaki Nakagawa, Yuichi Togashi

**Affiliations:** ^1^Department of Mathematical and Life Sciences, Graduate School of Science, Hiroshima UniversityHigashi-Hiroshima, Japan; ^2^Research Center for the Mathematics on Chromatin Live Dynamics, Hiroshima UniversityHigashi-Hiroshima, Japan

**Keywords:** small-number issues, catalytic reaction networks, chemical master equations, probability generating functions, systems biology, mathematical biology, population dynamics

## Abstract

Cell activities primarily depend on chemical reactions, especially those mediated by enzymes, and this has led to these activities being modeled as catalytic reaction networks. Although deterministic ordinary differential equations of concentrations (rate equations) have been widely used for modeling purposes in the field of systems biology, it has been pointed out that these catalytic reaction networks may behave in a way that is qualitatively different from such deterministic representation when the number of molecules for certain chemical species in the system is small. Apart from this, representing these phenomena by simple binary (on/off) systems that omit the quantities would also not be feasible. As recent experiments have revealed the existence of rare chemical species in cells, the importance of being able to model potential small-number phenomena is being recognized. However, most preceding studies were based on numerical simulations, and theoretical frameworks to analyze these phenomena have not been sufficiently developed. Motivated by the small-number issue, this work aimed to develop an analytical framework for the chemical master equation describing the distributional behavior of catalytic reaction networks. For simplicity, we considered networks consisting of two-body catalytic reactions. We used the probability generating function method to obtain the steady-state solutions of the chemical master equation without specifying the parameters. We obtained the time evolution equations of the first- and second-order moments of concentrations, and the steady-state analytical solution of the chemical master equation under certain conditions. These results led to the rank conservation law, the connecting state to the winner-takes-all state, and analysis of 2-molecules *M*-species systems. A possible interpretation of the theoretical conclusion for actual biochemical pathways is also discussed.

## 1. Introduction

Biochemical systems consist of a variety of chemicals, including proteins, nucleic acids, and also small metabolites. Enzymatic reactions, which play an important role in catalyzing many biological reactions, are particularly important to maintain the structure and activity of these systems. Hence, biochemical systems are often modeled as catalytic reaction networks.

These networks are typically analyzed by using deterministic ordinary differential equations with respect to concentrations of chemical species, so-called reaction rate equations [or partial differential equations (reaction-diffusion equations) for spatially distributed, non-uniform cases]; i.e., the concentrations are represented by continuous variables. However, because each chemical actually consists of molecules, the concentration of each species should be a discrete variable. The effects of such discreteness as well as finite-size fluctuations in stochastic reactions become non-negligible if the number of molecules in the system is small. In theory, situations such as these can result in phenomena that cannot be described by rate equations (as well as those equations with additional noise; Togashi and Kaneko, 2001, 2007; Awazu and Kaneko, 2007, 2009).

In contrast, gene regulations are often modeled as a combination of binary (on/off) switches, typically represented by Boolean network models (Kauffman, 1969). However, this approach does not consider the quantities of chemicals such as DNA, mRNA, and proteins. Even for the seemingly digital expression of genes, a stoichiometry involving DNA cannot be ignored, as seen in X-chromosome inactivation (2 to 1) and the trisomy syndrome (2 to 3). Thus, the use of a binary (on/off) representation would also be inappropriate. We therefore need to consider the number of molecules.

Recent experiments have shown the existence in the cell of proteins that only consist of a few molecules each (Taniguchi et al., 2010), and these potential biological phenomena (sometimes referred to as the *small-number issue*) have been gradually recognized by biologists. General theories that would enable predictions in small number situations would be helpful to every biological scientist seeking to understand biochemical processes on any level. Of course, the distributional behavior of such discrete stochastic systems is described by the chemical master equation. However, it is generally difficult to obtain its solution; hence, most efforts have been devoted to the development of approximation and simulation methods and their application (Gillespie, 1976, 1977; Munsky and Khammash, 2006; Lee et al., 2009; Kim and Lee, 2012).

The effects of small numbers in particular systems, e.g., small autocatalytic systems have been mathematically analyzed (Ohkubo et al., 2008; Biancalani et al., 2012, 2014; Houchmandzadeh and Vallade, 2015; Saito and Kaneko, 2015). In addition, a parameter representing the degree of discreteness in the number of molecules has been introduced (Haruna, 2010, 2015). In this work, we pursue analytical frameworks for studying the effects of small numbers in catalytic reaction networks by following an approach that is as general as possible. Our aim is to obtain the steady-state analytical solution for the chemical master equation without specifying parameters, rather than developing numerical schemes to solve it as was previously done (Kim and Lee, 2012). Furthermore, we try to theoretically describe the long-term behavior of the system by only using information about relationships between elements, thereby implying that we aim to produce results that could be applied to studies in any field.

Our framework provides good operability because our formulas have a specific and satisfyingly simple form, and enables us to obtain the steady state for a wide class of catalytic reaction networks because our framework never specifies any parameters for the networks. We use a probability generating function approach. The probability generating function approach to stochastic chemical kinetics itself has been proposed a long time ago (e.g., Krieger and Gans, 1960; McQuarrie, 1967), after which it spread to biological stochastic kinetics studies (e.g., stochastic gene expression Thattai and van Oudenaarden, 2001; Shahrezaei and Swain, 2008); thus, physicists as well as chemists and mathematical biologists are familiar with the approach. Our main contributions in this paper relate to the efficient usage of the probability generating function (as in Gadgil et al., 2005 regarding first-order reaction networks). Therefore, our approach for obtaining the steady state is understandably easier than that followed in a previous study (Anderson et al., 2010) while, as a consequence, our results are consistent with that study (see Theorem 4.1 and 4.2 in Anderson et al., 2010). Furthermore, since our method uses a procedure based on analytical calculations, it can be easily converted to a computer algorithm.

The present paper is organized as follows. In Section 2.1, we define the catalytic reaction network considered in this paper. The chemical master equation (CME) is provided in Section 2.2. We introduce the probability generating function (PGF) and derive the generating function equation (GFE) in Section 2.3. In subsequent Sections (2.3.1–2.3.3), we show that the GFE introduces the time evolution equation of the first-order and second-order moments of concentrations (we refer to the first-order moment time evolution equation as the pre-rate equation, PRE), and the second-order moment expression of time-averaged concentrations (SME). Section 2.4 is devoted to obtaining the steady-state solutions of the GFE. To simplify the GFE, we neglect the non-catalytic reactions considered as perturbation for the catalytic reaction network if the system is “entirely ergodic.” In Section 2.4.2.3 as the main result, we obtain the probability generating function without winner-takes-all states (PGFwoWTAS) including the solutions of the corresponding rate equation. In Section 3, we describe applications of these results: the rank conservation law, the connecting state to the winner-takes-all state, analysis of 2-molecules *M*-species systems, and non-autocatalyzation of autocatalytic reaction networks. The prospects of our theory in terms of the small-number issue are briefly discussed in Section 4.

## 2. Methods and results

### 2.1. Catalytic reaction networks

Consider an abstract catalytic reaction network consisting of *M* chemical species and *N* molecules in a well-stirred reactor of volume *V*, as in Awazu and Kaneko (2007). Each species is labeled by each of the integers between 1 and *M*. The total number of molecules *N* is always conserved in reaction processes. This chemical reaction system involves both catalytic reactions and non-catalytic reactions, to prevent catalytic reactions from stopping:

- Catalytic reactions (two-body catalysis):
  (1) $$\begin{array}{ll} i\overset{j}{\to }k, & \; \end{array}$$
  where the species *i, j, k* ∈ [1, *M*] represent a substrate, a catalyst, and a product. The reaction rate constant is represented by *R*_*ijk*_ > 0. If this catalytic reaction does not exist, we specify *R*_*ijk*_ = 0. Therefore, the catalytic reaction networks are determined by *R*_*ijk*_. In this paper, we impose the following conditions for the catalytic reaction networks;
  - *R*_*iik*_ = 0; Substrate ≠ Catalyst.
  - *R*_*iji*_ = 0; Substrate ≠ Product.
  - *R*_*ikk*_ = 0; Autocatalytic reactions are not included.
  - #{*k* : *R*_*ijk*_ > 0 (∀*i, j*)} = 1;
  - One product against a substrate and a catalyst.
- Non-catalytic reactions (one-body reactions):
  (2) $$\begin{array}{l} i\overset{\text{Prob. }1/M}{\to }j. \end{array}$$
  This reaction exists for all combinations between each species, but a product *j* ∈ [1, *M*] is uniformly-randomly selected from all species 1 to *M*. The rate constant is set ε > 0 in common, where ε is very small, i.e., ε ≪ min{*R*_*ijk*_ > 0}.

The state of this catalytic reaction network is specified by the combination of *M* natural numbers *n* = (*n*_1_, *n*_2_, ⋯ , *n*_*M*_), where *n*_*i*_ ∈ [0, *N*] is the number of molecules of the *i*th-species. For later convenience, we introduce the following notations: the *state space of the catalytic reaction network W*_*M, N*_ (abbr. *W*) is represented by

(3) $$\begin{array}{ll} W:=\left\{n\in {\left[0,N\right]}^{M}|n_{1}+\cdots +n_{M}=N\right\}, & \; \end{array}$$

which consists of $\left(\left(\frac{M}{N}\right)\right):=\frac{(N+M-1)!}{(M-1)!N!}$ points; a *collection consisting of (one species) winner-takes-all states I*_*M, N*_ (abbr. *I*) is represented by

(4) $$\begin{array}{ll} I:=\left\{n\in W_{M,N}|\exists i\in \left[1,M\right]\text{ s.t.}n_{i}=N\right\}, & \; \end{array}$$

which consists of *M* points. Of course, the winner*s*-take-all states of more than one species can be considered, but we focus on the winner-takes-all states of one species by supposing the system satisfies a certain condition, *entire ergodicity* (see Section 2.4.2.4).

In the present paper, we are interested in the *N*-dependence of the concentration of each species *x*_*i*_ = *n*_*i*_/*V*. We basically consider a situation in which the total density of molecules ρ = *N*/*V* is conserved, even if the total number of molecules *N* is changed.

### 2.2. The chemical master equation

The rate constant *R*_*ijk*_ in the catalytic reaction is defined as the number of reactions per unit volume, unit concentration, and unit time. Therefore, the number of reactions per unit time in the catalytic reaction $i\overset{j}{\to }k$, such that the concentrations are *x*_*i*_ and *x*_*j*_, is

(5) $$\begin{array}{ll} R_{ijk}x_{i}x_{j}V=R_{ijk}\frac{n_{i}n_{j}}{V}=R_{ijk}\frac{n_{i}n_{j}}{N}\rho , & \; \end{array}$$

where ρ = *N*/*V* is the total density of molecules in the vessel. On the other hand, the number of reactions per unit time in the non-catalytic reaction *i* → *j* with probability 1/*M* is

(6) $$\begin{array}{ll} \varepsilon x_{i}V\times \frac{1}{M}=\frac{\varepsilon }{M}n_{i}. & \; \end{array}$$

Then, the time-evolution of the probability *P*(***n**, t*), with which the system is in the state ***n*** at time *t*, obeys the chemical master equation (CME):

(7) $$\begin{array}{l} \frac{dP\left(n,t\right)}{dt}=\frac{\rho }{N}\underset{i,j,k}{\sum }R_{ijk}\left(E_{i}^{+1}E_{k}^{-1}-1\right)n_{i}n_{j}P\left(n,t\right)\\ \;+\frac{\varepsilon }{M}\underset{i,j}{\sum }\left(E_{i}^{+1}E_{j}^{-1}-1\right)n_{i}P\left(n,t\right), \end{array}$$

where $E_{i}^{\pm m}$ are step operators, i.e.,

(8) $$\begin{array}{ll} E_{i}^{\pm m}f\left(n_{1},\cdots \;,n_{i},\cdots \;,n_{M}\right):=f\left(n_{1},\cdots \;,n_{i}\pm m,\cdots \;,n_{M}\right). & \; \end{array}$$

Of course, the state space on which the probability *P*(·, *t*) is supported, is the previously described *W*_*M, N*_.

### 2.3. Probability generating function method

The probability generating function (PGF) is useful to analyze the CME:

(9) $$\phi (z,t):=\sum_{n_{1},\cdots ,n_{M}}P(n,t)\;z_{1}{}^{n_{1}}\cdots z_{M}{}^{n_{M}}.$$

Note that the following expressions are translated to differential forms of the PGF:

(10a) $$\begin{array}{lll} n_{i}P\left(n,t\right) & \mapsto z_{i}\frac{\partial }{\partial z_{i}}\phi \left(z,t\right),\; & \; \end{array}$$

(10b) $$\begin{array}{lll} n_{i}n_{j}P\left(n,t\right) & \mapsto z_{i}z_{j}\frac{\partial }{\partial z_{i}}\frac{\partial }{\partial z_{j}}\phi \left(z,t\right)\;\left(i\neq j\right),\; & \; \end{array}$$

(10c) $$\begin{array}{lll} E_{i}^{+1}E_{j}^{-1}n_{i}P\left(n,t\right) & \mapsto z_{j}\frac{\partial }{\partial z_{i}}\phi \left(z,t\right)\;\left(i\neq j\right),\; & \; \end{array}$$

(10d) $$\begin{array}{lll} E_{i}^{+1}E_{k}^{-1}n_{i}n_{j}P\left(n,t\right) & \mapsto z_{j}z_{k}\frac{\partial }{\partial z_{i}}\frac{\partial }{\partial z_{j}}\phi \left(z,t\right)\;\left(i\neq j\neq k\neq i\right).\; & \; \end{array}$$

Therefore, rewriting the CME to the equation governing the PGF enables the generating function equation (GFE) to be obtained:

(11) $$\begin{array}{l} \frac{\partial \phi \left(z,t\right)}{\partial t}=\frac{\rho }{N}\underset{i,j,k}{\sum }R_{ijk}\left(z_{k}-z_{i}\right)\;z_{j}\frac{\partial }{\partial z_{i}}\frac{\partial }{\partial z_{j}}\phi \left(z,t\right)\\ \;+\frac{\varepsilon }{M}\underset{i,j}{\sum }\left(z_{i}-z_{j}\right)\frac{\partial }{\partial z_{j}}\phi \left(z,t\right). \end{array}$$

The GFE consists of continuous variables, unlike the CME, which consists of discrete variables.

Once we obtain the PGF ϕ as a solution to the GFE, we can derive all statistics of the catalytic reaction network; for example, the ensemble averages (first-order moments) and second-order moments become

(12a) $$\begin{array}{l} \langle n_{i}\rangle (t)={\frac{\partial }{\partial z_{i}}\phi (z,t)|}_{z=1}, \end{array}$$

(12b) $$\begin{array}{l} \langle n_{i}n_{j}\rangle (t)={\frac{\partial^{2}}{\partial z_{i}\partial z_{j}}\phi (z,t)|}_{z=1}\;(i\neq j), \end{array}$$

(12c) $$\begin{array}{l} \langle n_{l}(n_{l}-1)\rangle (t)={\frac{\partial^{2}}{\partial z_{l}^{2}}\phi (z,t)|}_{z=1}, \end{array}$$

and the marginal distributions become

(13) $$p_{i}(n,t)=\frac{1}{n!}{\frac{\partial^{n}}{\partial z_{i}^{n}}\phi (z,t)|}_{z_{i}=0;\;z_{j}=1(j\neq i)}\;(0\leq n\leq N),$$

where *p*_*i*_(*n, t*) is defined by

(14) $$p_{i}(n,t):=\sum_{\begin{array}{c} n_{1},\cdots ,n_{M}\\ \left(\text{except }n_{i}\right) \end{array}}{P(n,t)|}_{n_{i}=n}.$$

#### 2.3.1. The pre-rate equation

Rate equations are differential equations for the concentrations *x*_*i*_ of chemical species *i* = 1, ⋯ , *M* when *N* → ∞. We use the GFE to derive a formula, which is reminiscent of the rate equation.

Differentiating both sides of Equation (11) by *z*_*i*_, substituting ***z*** = ***1***, and writing *x*_*i*_ = *n*_*i*_/*V*, leads to the following pre-rate equation (PRE);

(15) $$\begin{array}{l} \frac{d}{dt}\langle x_{i}\rangle =\underset{j,k}{\sum }\left(R_{jki}\langle x_{j}x_{k}\rangle -R_{ikj}\langle x_{i}x_{k}\rangle \right)+\varepsilon \left(\frac{\rho }{M}-\langle x_{i}\rangle \right).\; \end{array}$$

Note that, as we are considering a system of which the total number of molecules *N* is conserved, the concentration conservation law (CCL) must be satisfied;

(16) $$\begin{array}{l} \underset{i}{\sum }\langle x_{i}\rangle \left(t\right)=\rho \;\left(\forall t\right),\; \end{array}$$

where the PRE (Equation 15) is consistent with the CCL (Equation 16).

If the independence 〈*x*_*i*_*x*_*j*_〉 = 〈*x*_*i*_〉〈*x*_*j*_〉 (*i* ≠ *j*) holds, the PRE (Equation 15) becomes the same expression as the rate equation. On the other hand, the PRE does not explicitly include extensive variables, such as the total number of molecules *N*. Therefore, the formula (Equation 15) always holds for an arbitrary molecular number *N* unless the total molecular density ρ is changed.

#### 2.3.2. Second-order moment expression for time-averaged concentrations

We suppose the following ergodicity to replace ensemble-averages with time-averages; ${\bar{n}}_{i}={\langle n_{i}\rangle }_{*}$ i.e.,

(17) $$\begin{array}{l} \underset{T\to \infty }{\lim}\frac{1}{T}\int_{0}^{T}n_{i}\left(t\right)dt=\underset{n_{1},\cdots \;,n_{M}}{\sum }n_{i}P_{*}\left(n\right),\; \end{array}$$

where P_*_(***n***) is the steady-state solution of the CME corresponding to almost all initial conditions. Considering the steady state in the PRE [that is, taking *t* → ∞ on both sides of Equation (15)], one obtains the following second-order moment expression (SME) for time-averaged concentrations:

(18) $$\begin{array}{l} {\bar{x}}_{i}=\frac{\rho }{M}+\frac{1}{\varepsilon }\underset{j,k}{\sum }\left(R_{jki}\bar{x_{j}x_{k}}-R_{ikj}\bar{x_{i}x_{k}}\right).\; \end{array}$$

The second term on the right hand side of Equation (18) represents the difference from the uniform concentration ρ/*M*.

If the independence $\bar{x_{i}x_{j}}={\bar{x}}_{i}{\bar{x}}_{j}$ holds, Equation (18) becomes an equation that only includes the concentrations ${\bar{x}}_{i}$. Therefore, in combination with the time-averaged version of the CCL (Equation 16), the concentrations ${\bar{x}}_{i}$ can be determined without including unknown quantities. However, the actual concentrations ${\bar{x}}_{i}$ depend on their second-order moments $\bar{x_{i}x_{j}}$.

#### 2.3.3. Time-evolution of second-order moments

Determining the concentrations ${\bar{x}}_{i}$ from the SME (Equation 18) requires us to know their second-order moments $\bar{x_{i}x_{j}}$.

Differentiating both sides of the GFE (Equation 11) by *z*_*l*_ and *z*_*m*_ (*l* ≠ *m*), substituting ***z*** = ***1***, and writing *x*_*i*_ = *n*_*i*_/*V*, then, after simplification, the following time-evolution equation of second-order moments (TESM) is obtained:

(19a) $$\begin{array}{l} \frac{d}{dt}\langle x_{l}x_{m}\rangle =-\frac{\rho }{N}\sum_{i}(R_{lim}\langle x_{i}x_{l}\rangle +R_{mil}\langle x_{i}x_{m}\rangle )\\ \;+\sum_{i,j}\{R_{ijl}\langle x_{i}x_{j}x_{m}\rangle +R_{ijm}\langle x_{i}x_{j}x_{l}\rangle -(R_{lij}+R_{mij})\\ \;\langle x_{i}x_{l}x_{m}\rangle \}\\ \;+\frac{\varepsilon }{M}\left\{\frac{N-1}{N}\rho (\langle x_{l}\rangle +\langle x_{m}\rangle )-2M\langle x_{l}x_{m}\rangle \right\}\\ \;(1\leq l<m\leq M). \end{array}$$

On the other hand, twice differentiating both sides of the GFE (Equation 11) by *z*_*l*_, substituting ***z*** = ***1***, and writing *x*_*i*_ = *n*_*i*_/*V*, then after some simplification, the following alternative TESM is obtained:

(19b) $$\begin{array}{l} \frac{d}{dt}\langle x_{l}\left(x_{l}-\frac{\rho }{N}\right)\rangle =2\underset{i,j}{\sum }\left(R_{ijl}\langle x_{i}x_{j}x_{l}\rangle -R_{lij}\langle x_{i}x_{l}\left(x_{l}-\frac{\rho }{N}\right)\rangle \right)\;\\ \;+\frac{2\varepsilon }{M}\left\{\frac{N+M-1}{N}\rho \langle x_{l}\rangle -M\langle x_{l}^{2}\rangle \right\}\;\\ \;\left(1\leq l\leq M\right).\; \end{array}$$

The expression of TESMs (Equation 19) consists of *M*(*M*+1)/2 equations containing the third-order moments 〈*x*_*i*_*x*_*j*_*x*_*k*_〉(*t*). Therefore, the TESMs are not effective unless the systems are restricted such that the third-order moments vanish (e.g., 2-molecules systems). If *N* = 2, because $\partial_{z_{i}}$$\partial_{z_{j}}$$\partial_{z_{k}}$ϕ = 0 (ϕ is a second-order polynomial), one can calculate the 2-molecules version of the TESMs (2mTESMs);

(20a) $$\begin{array}{l} \frac{d}{dt}\langle x_{l}x_{m}\rangle =\frac{\rho }{2}\sum_{i}\{R_{iml}\langle x_{i}x_{m}\rangle +R_{ilm}\langle x_{i}x_{l}\rangle \\ \;-(R_{lmi}+R_{mli})\langle x_{l}x_{m}\rangle \}\\ \;+\frac{\rho \varepsilon }{2M}(\langle x_{l}\rangle +\langle x_{m}\rangle )-2\varepsilon \langle x_{l}x_{m}\rangle \\ \;(1\leq l<m\leq M), \end{array}$$

(20b) $$\begin{array}{l} \frac{d}{dt}\langle x_{l}\left(x_{l}-\frac{\rho }{N}\right)\rangle =\frac{2\varepsilon }{M}\left(\frac{M+1}{2}\rho \langle x_{l}\rangle -M\langle x_{l}^{2}\rangle \right)\;\left(1\leq l\leq M\right).\; \end{array}$$

Treatment of the 2mTESMs (Equation 20) to demonstrate their effectiveness appears in a later section.

### 2.4. Steady-state solutions of GFE

If the GFE (Equation 11) can be solved, this would enable us to obtain all the statistics of the catalytic reaction networks. However, it is generally difficult to solve. Here, we focus on the steady-state solutions of the GFE and consider the case that the ε-term in the GFE can be ignored. Through the following discussion, we see that the approximation is effective only if the system is ergodic.

#### 2.4.1. The case of non-catalytic reactions only

First, we consider the steady-state solutions of non-catalytic reactions only as an introduction. The PGF $\phi_{*}^{\text{nc}}\left(z\right)$, corresponding to the steady state in the case of non-catalytic reactions only, should satisfy the following equation:

(21) $$\begin{array}{l} 0=\underset{i,j}{\sum }\left(z_{i}-z_{j}\right)\frac{\partial }{\partial z_{j}}\phi_{*}^{\text{nc}}\left(z\right).\; \end{array}$$

By exchanging the subscripts *i* and *j* in the second term, one obtains

(22) $$\begin{array}{l} 0=\underset{i,j}{\sum }z_{i}\left(\frac{\partial \phi_{*}^{\text{nc}}}{\partial z_{j}}-\frac{\partial \phi_{*}^{\text{nc}}}{\partial z_{i}}\right).\; \end{array}$$

Because the coefficients of variables *z*_*i*_ must be zero, $\phi_{*}^{\text{nc}}$ must satisfy the following equations:

(23) $$\begin{array}{l} 0=\underset{j}{\sum }\left(\frac{\partial \phi_{*}^{\text{nc}}}{\partial z_{j}}-\frac{\partial \phi_{*}^{\text{nc}}}{\partial z_{i}}\right)\;\left(\forall i\right),\; \end{array}$$

equivalently,

(24) $$\begin{array}{l} \frac{\partial \phi_{*}^{\text{nc}}}{\partial z_{i}}=\frac{1}{M}\overset{M}{\underset{j=1}{\sum }}\frac{\partial \phi_{*}^{\text{nc}}}{\partial z_{j}}\;\left(\forall i\right).\; \end{array}$$

Equation (24) implies that all of $\partial_{z_{i}}\phi_{*}^{\text{nc}}$ (*i* = 1, ⋯ , *M*) are equal to each other, i.e.,

(25) $$\begin{array}{l} \frac{\partial \phi_{*}^{\text{nc}}}{\partial z_{i}}=\frac{\partial \phi_{*}^{\text{nc}}}{\partial z_{j}}\;\left(1\leq i<j\leq M\right).\; \end{array}$$

Considering that the PGF $\phi_{*}^{\text{nc}}\left(z\right)$ is an *N*th order polynomial of *z*_*i*_ and must satisfy the condition $\phi_{*}^{\text{nc}}\left(1\right)=1$ by definition, the following solution of Equation (25) can be found:

(26) $$\begin{array}{l} \phi_{*}^{\text{nc}}\left(z\right)={\left(\frac{z_{1}+z_{2}+\cdots +z_{M}}{M}\right)}^{N}.\; \end{array}$$

Therefore, we obtain the following stationary distribution in the case of non-catalytic reactions only:

(27) P*nc(n)=1MN(Nn1,n2,⋯,nM)  (n∈W),  

where (Nn1,n2,⋯,nM):=N!n1!n2!⋯nM! are multinomial coefficients. Furthermore, Equation (13) can also be used to derive the marginal distribution of the *i*-th species:

(28) pi*nc(n)=(Nn)(1M)n(1-1M)N-n     (0≤n≤N),  

which is the binomial distribution with parameters *N* and 1/*M*.

If we suppose the ergodicity $\langle n_{i}\rangle ={\bar{n}}_{i}$, the following statistics can be calculated from Equation (12):

(29a) $$\begin{array}{l} {\bar{x}}_{i}=\frac{\rho }{M}\;\left(i=1,\cdots \;,M\right),\; \end{array}$$

(29b) $$\begin{array}{l} \bar{x_{i}x_{j}}={\left(\frac{\rho }{M}\right)}^{2}\left(1-\frac{1}{N}\right)\;\left(i\neq j\right),\; \end{array}$$

(29c) $$\begin{array}{l} \text{Var}\left[x_{i}\right]={\left(\frac{\rho }{M}\right)}^{2}\frac{M-1}{N}\;\left(i=1,\cdots \;,M\right),\; \end{array}$$

where $\text{Var}\left[x_{i}\right]:=\bar{x_{i}^{2}}-{\bar{x_{i}}}^{2}$ is the variance of the concentration *x*_*i*_ = *n*_*i*_/*V*.

#### 2.4.2. The case of catalytic reactions only

Next, we consider the steady-state solutions of catalytic reactions only, assuming that the ε-term in the GFE (Equation 11) can be ignored. The steady-state solutions are assumed to have a form similar to Equation (26), including undetermined coefficients (λ_*i*_) deriving from the network structure (*R*_*ijk*_).

##### 2.4.2.1. A condition for finding the steady state: λ-condition

The PGF $\phi_{*}^{\text{c}}\left(z\right)$, corresponding to the steady state in the case of catalytic reactions only, should satisfy the following equation;

(30) $$\begin{array}{l} 0=\underset{i,j,k}{\sum }R_{ijk}\left(z_{k}-z_{i}\right)z_{j}\frac{\partial }{\partial z_{i}}\frac{\partial }{\partial z_{j}}\phi_{*}^{\text{c}}\left(z\right).\; \end{array}$$

Construction of the steady-state solution requires us to find a particular solution of Equation (30) as bases of linear space consisting of *N*-th order polynomials. Accordingly, let us assume the following extended form of Equation (26) by introducing parameters ${\left\{\lambda_{i}\right\}}_{i=1}^{M}$, which eventually correspond to the concentrations (per total density ρ) of each species in the continuous limit *N* → ∞ as shown in Section 2.4.2.3:

(31) $$\begin{array}{l} \phi_{*}^{\text{c}}\left(z\right)={\left(\lambda_{1}z_{1}+\lambda_{2}z_{2}+\cdots +\lambda_{M}z_{M}\right)}^{N},\; \end{array}$$

where

(32) $$\begin{array}{l} \overset{M}{\underset{i=1}{\sum }}\lambda_{i}=1\;\left(0\leq \lambda_{1},\lambda_{2}\cdots \;,\lambda_{M}\leq 1\right),\; \end{array}$$

because ϕ_*_(1) = 1. Substituting Equation (31) into Equation (31) and setting the coefficients of variables *z*_*i*_*z*_*j*_ as zero, gives the following condition for {λ_*i*_λ_*j*_}:

(33) $$\begin{array}{l} \overset{M}{\underset{k=1}{\sum }}\left\{R_{kij}\lambda_{k}\lambda_{i}+R_{kji}\lambda_{k}\lambda_{j}-\left(R_{ijk}+R_{jik}\right)\lambda_{i}\lambda_{j}\right\}=0\\ \;\left(1\leq i<j\leq M\right).\; \end{array}$$

The λ-condition (Equation 33) represents *M*(*M* − 1)/2 homogeneous equations for λ_*i*_λ_*j*_; therefore, λ_*i*_ can be calculated by combining the λ-condition (Equation 33) with Equation (32). The λ-condition has trivial solutions:

(34) $$\begin{array}{l} \left(\lambda_{1},\cdots \;,\lambda_{i},\cdots \;,\lambda_{M}\right)=\left(0,\cdots \;,0,1,0,\cdots \;,0\right)\;\exists i\in \left[1,M\right].\; \end{array}$$

These solutions represent the states for which the *i*-th species take all molecules (the *winner-takes-all state*). On the other hand, there is also a non-trivial solution. In the following paragraph, we treat three-species systems (*M* = 3) to demonstrate that non-trivial solutions do exist. The following procedures can easily be extended to those for arbitrary *M* species systems.

##### 2.4.2.2. Demonstration for non-trivial solutions of the λ-condition

The λ-condition (Equation 33) can be rewritten in matrix form; i.e., in the case of *M* = 3 for example,

(35) $$\left[\begin{array}{ccc} -R_{123}-R_{213} & -R_{312} & R_{321}\\ R_{213} & -R_{132}-R_{312} & R_{231}\\ R_{123} & R_{132} & -R_{231}-R_{321} \end{array}\right]\left[\begin{array}{ll} \lambda_{1} & \lambda_{2}\\ \lambda_{1} & \lambda_{3}\\ \lambda_{2} & \lambda_{3} \end{array}\right]=0$$

The 3 × 3 matrix (*A*) on the left-hand side can be rewritten in terms of its column vectors:

(36) $$A=\begin{array}{c} {}[-R_{123}e_{1}-R_{213}e_{2},R_{312}e_{2}-R_{132}e_{3},R_{321}e_{1}+R_{231}e_{3} \end{array}],$$

where ${e_{1}}^{T}=\left(1,0,-1\right)$, ${e_{2}}^{T}=\left(1,-1,0\right)$, and ${e_{3}}^{T}=\left(0,1,-1\right)$. According to the determinant property, i.e., det[***a, b, c***] = −det[***b, a, c***], one has

(37) $$\begin{array}{l} \det\left(A\right)=0.\; \end{array}$$

Therefore, the non-trivial solution of Equation (35), i.e., the eigenvector corresponding to the eigenvalue 0 of the matrix *A*, certainly exists and it has the following expression:

(38) $$\left[\begin{array}{c} \lambda_{1}\lambda_{2}\\ \lambda_{1}\lambda_{3}\\ \lambda_{2}\lambda_{3} \end{array}\right]\propto \left[\begin{array}{c} R_{312}R_{231}+R_{321}R_{132}+R_{312}R_{321}\\ R_{213}R_{321}+R_{231}R_{123}+R_{213}R_{231}\\ R_{123}R_{312}+R_{132}R_{213}+R_{123}R_{132} \end{array}\right]\equiv \left[\begin{array}{c} \Lambda_{3}\\ \Lambda_{2}\\ \Lambda_{1} \end{array}\right].$$

The proportionality constant (> 0) can be determined by the condition (Equation 32), and thus the desired non-trivial solution of the λ-condition (Equation 33) for *M* = 3 is obtained:

(39a) $$\begin{array}{l} \lambda_{1}=\frac{\Lambda_{2}\Lambda_{3}}{\Lambda_{1}\Lambda_{2}+\Lambda_{1}\Lambda_{3}+\Lambda_{2}\Lambda_{3}},\; \end{array}$$

(39b) $$\begin{array}{l} \lambda_{2}=\frac{\Lambda_{1}\Lambda_{3}}{\Lambda_{1}\Lambda_{2}+\Lambda_{1}\Lambda_{3}+\Lambda_{2}\Lambda_{3}},\; \end{array}$$

(39c) $$\begin{array}{l} \lambda_{3}=\frac{\Lambda_{1}\Lambda_{2}}{\Lambda_{1}\Lambda_{2}+\Lambda_{1}\Lambda_{3}+\Lambda_{2}\Lambda_{3}}.\; \end{array}$$

Note that it is not guaranteed that the above solution always represents a non-trivial solution of the λ-condition (Equation 33). For example, the case of Λ_1_ = 0 (where Λ_2_ and Λ_3_ are not zero) implies the existence of a trivial solution (λ_1_, λ_2_, λ_3_) = (1, 0, 0). In another example, the case of Λ_1_ = Λ_2_ = 0 implies that the denominator becomes zero; thus, the expression becomes indefinite.

##### 2.4.2.3. PGF without winner-takes-all states

We are interested in those states in which any species does not take all molecules, because the actual simulations are performed by using the initial states excluding the winner-takes-all states. The PGF without the winner-takes-all states is represented by a linear summation of winner-takes-all states $z_{i}^{N}$ and Equation (31) if the λ-condition has a non-trivial solution like Equation (39);

(40) $$\begin{array}{l} \phi_{*}^{\text{c}}\left(z\right)=\overset{M}{\underset{i=1}{\sum }}b_{i}z_{i}^{N}+b_{M+1}{\left(\overset{M}{\underset{i=1}{\sum }}\lambda_{i}z_{i}\right)}^{N},\; \end{array}$$

where *b*_1_+⋯+*b*_*M*+1_ = 1. The *take-all exclusion conditions* are that the coefficients of $z_{i}^{N}$ are zero;

(41) $$\begin{array}{l} b_{i}+b_{M+1}\lambda_{i}^{N}=0\;\left(i=1,2,\cdots \;,M\right).\; \end{array}$$

Therefore, we obtain the desired PGF without the winner-takes-all states (PGFwoWTAS);

(42) $$\begin{array}{l} \phi_{*}^{\text{c}}\left(z\right)=\frac{{\left(\sum_{i=1}^{M}\lambda_{i}z_{i}\right)}^{N}-\sum_{i=1}^{M}{\left(\lambda_{i}z_{i}\right)}^{N}}{1-\sum_{i=1}^{M}\lambda_{i}^{N}},\;\\ \;\overset{M}{\underset{i=1}{\sum }}\lambda_{i}=1,\;0\leq \lambda_{i}<1,\; \end{array}$$

which immediately implies the following stationary distribution in the case of catalytic reactions only;

(43) P*c(n)={(Nn1,n2,⋯ ,nM)∏​i=1Mλini1−∑​i=1MλiN     (n∈W\I),0                                                         (n∈I), 

where $\left(\begin{array}{l} N\\ n_{1},\;n_{2},\cdots ,n_{M} \end{array}\right):=\frac{N!}{n_{1}!n_{2}!\cdots n_{M}!}$ are multinomial coefficients. Furthermore, we can calculate the marginal distribution of the *i*-th species from Equation (13);

(44) pi*c(n)={(1−λi)N+λiN−∑​l=1MλlN1−∑​l=1MλlN     (n=0),(Nn)λin(1−λi)N−n1−∑​l=1MλlN     (1≤n≤N−1),0                                                   (n=N)  

If we suppose the ergodicity $\langle n_{i}\rangle ={\bar{n}}_{i}$, the time-averaged concentrations can be derived from Equation (12);

(45a) $$\begin{array}{l} {\bar{x}}_{i}=\frac{\lambda_{i}-\lambda_{i}^{N}}{1-\sum_{i=1}^{M}\lambda_{i}^{N}}\rho \;\left(i=1,\cdots \;,M\right).\; \end{array}$$

The above equations indicate that λ_*i*_ means the concentration per total density ρ in the continuous limit *N* → ∞, that is, λ_*i*_ should be the solution of the classical rate equation. We can also calculate the second-order moments $\bar{x_{i}x_{j}}$ and the variances $Var\left[x_{i}\right]:=\bar{x_{i}^{2}}-{{\bar{x}}_{i}}^{2}$ from Equation (12);

(45b) $$\begin{array}{l} \bar{x_{i}x_{j}}=\frac{\lambda_{i}\lambda_{j}}{1-\sum_{k=1}^{M}\lambda_{k}^{N}}\left(1-\frac{1}{N}\right)\rho^{2}\;\left(i\neq j\right).\; \end{array}$$

(45c) $$\begin{array}{l} \text{Var}\left[x_{i}\right]=\left[\frac{\lambda_{i}^{2}-\lambda_{i}^{N}-\frac{1}{N}\lambda_{i}\left(\lambda_{i}-1\right)}{1-\sum_{i=1}^{M}\lambda_{i}^{N}}-{\left(\frac{\lambda_{i}-\lambda_{i}^{N}}{1-\sum_{i=1}^{M}\lambda_{i}^{N}}\right)}^{2}\right]\rho^{2}\;\\ \;\left(i=1,\cdots \;,M\right),\; \end{array}$$

In the continuous limit *N* → ∞, the above Equations (45b) and (45c) become λ_*i*_λ_*j*_ and 0, respectively, that is, the concentrations become mutually independent without fluctuating variables.

**Figure 1:**
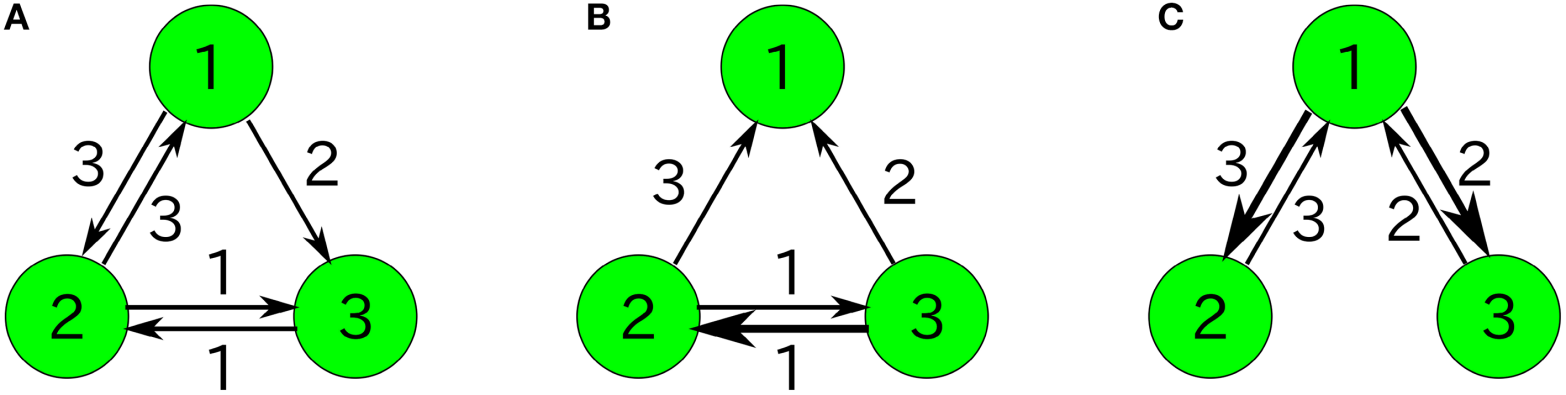
**Examples of catalytic reaction networks consisting of 3 species**. The rate constants are **(A)** *R*_123_ = 1, *R*_132_ = 1, *R*_213_ = 1, *R*_231_ = 1, *R*_312_ = 1, *R*_321_ = 0, **(B)** *R*_123_ = 0, *R*_132_ = 0, *R*_213_ = 1, *R*_231_ = 1, *R*_312_ = 2, *R*_321_ = 1, and **(C)** *R*_123_ = 1997/3, *R*_132_ = 1000/3, *R*_231_ = 1, *R*_321_ = 1, *R*_213_ = 0, *R*_312_ = 0.

We compare our formulas with simulation results that are obtained by applying the Gillespie algorithm (Gillespie, 1977) to the following three-species system (Figure 1A) as an example:

(46) $$\begin{array}{l} R_{123}=R_{132}=R_{213}=R_{231}=R_{312}=1,\;R_{321}=0;\;\rho =1,\; \end{array}$$

which implies the following by Equation (39):

(47) $$\begin{array}{l} \lambda_{1}=2/11,\;\lambda_{2}=3/11,\;\lambda_{3}=6/11.\; \end{array}$$

**Figure 2:**
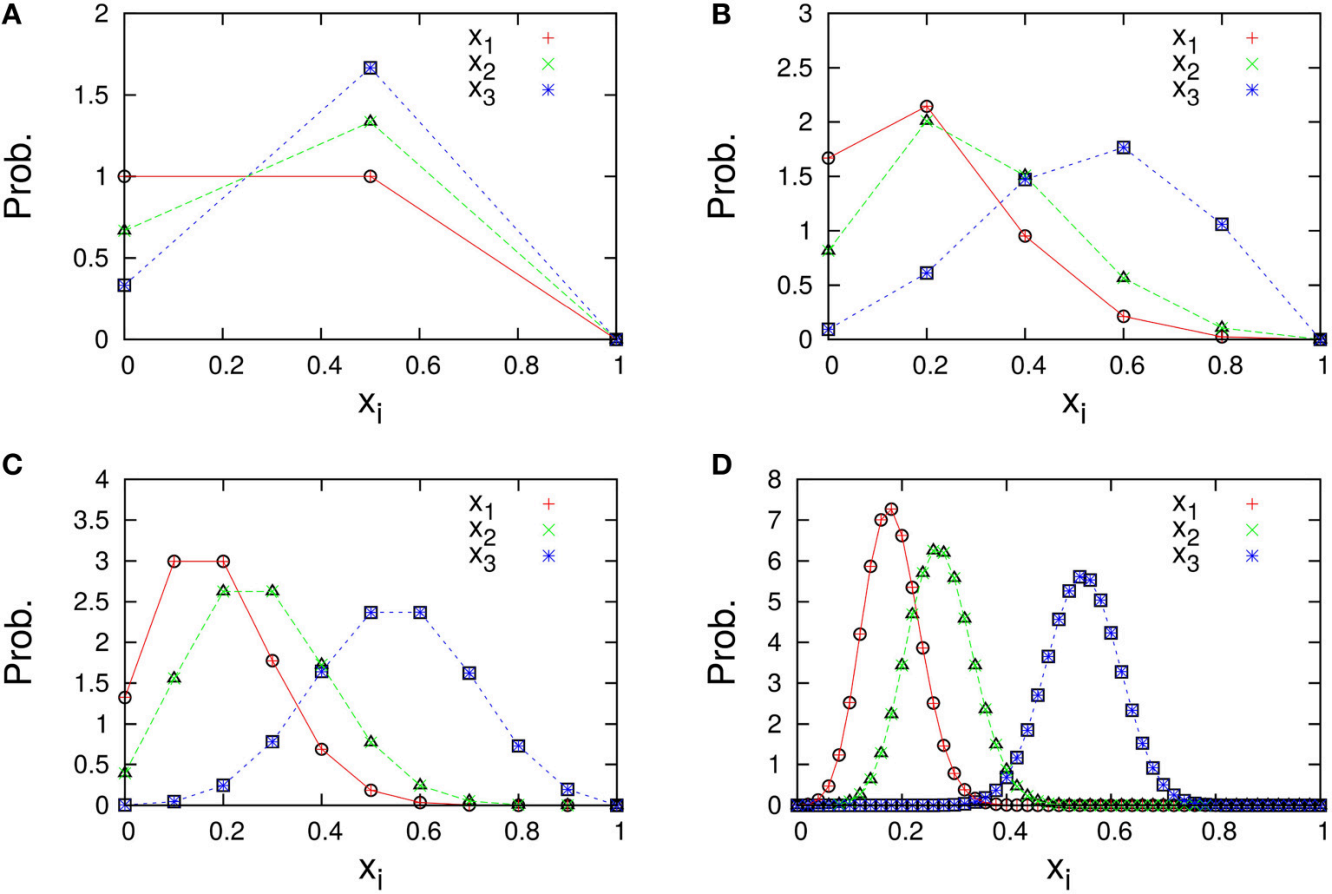
**Marginal distributions of ***x***_***i***_ = ***n***_***i***_/***N*** (***i*** = 1, 2, 3) in the three-species system of Figure 1A**. The total number of molecules in each figure is **(A)** *N* = 2, **(B)** *N* = 5, **(C)** *N* = 10, and **(D)** *N* = 50. Cross symbols (red, green, blue) represent the simulation results obtained numerically using the Gillespie algorithm (Gillespie, 1977), which is performed under the following condition; the total number of reactions: 10^8^, the number of reactions for transient exclusion: 10^7^, and the initial value (*n*_1_(0), *n*_2_(0), *n*_3_(0)) is randomly selected from *W* \ *I* such that the average per one-species is *N*/*M*. Empty symbols (circle, triangle, square) represent the theoretical expression $Np_{i*}^{\text{c}}\left(Nx\right)$ [Equation (44)] for λ_1_ = 2/11, λ_2_ = 3/11, and λ_3_ = 6/11.

**Figure 3:**
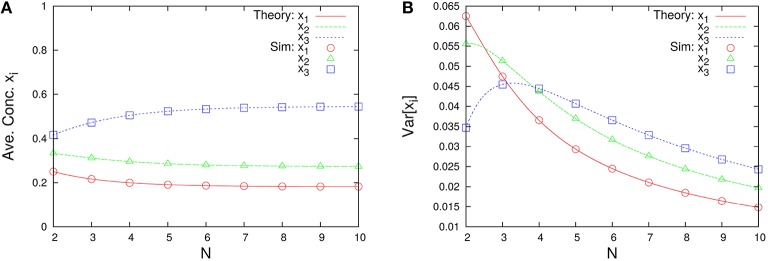
***N*-dependence of (A) the time-averaged concentrations ${\bar{x}}_{i}={\bar{n}}_{i}/N$ and (B) the variances of concentrations $\text{Var}\left[x_{i}\right]=\left(\bar{n_{i}^{2}}-{\bar{n}}_{i}^{2}\right)/N^{2}$ in the three-species system of Figure 1A**. Empty symbols are numerically obtained using the Gillespie algorithm, where the time averages are performed over a long time series *n*_*i*_(*t*) such that the total number of reactions reaches 10^8^. The initial values are randomly selected from *W* \ *I* such that the average per one-species is *N*/*M*, and the number of reactions for transient exclusion is 10^7^. Lines in each figure represent the theoretical expressions, Equations (45a) and (45c), for λ_1_ = 2/11, λ_2_ = 3/11, and λ_3_ = 6/11. One can see that the rank of concentrations is conserved but the rank of variances is exchanged between *N* = 2 and 4.

The marginal distributions are shown in Figure 2. Our formula Equation (44) is entirely in agreement with the simulation results. When the total number of molecules *N* is large, the marginal distributions can be approximated by normal distributions. Similarly, the formulas of the concentrations and the variances, Equations (45a) and (45c), are completely in agreement with the simulation results as shown in Figure 3. One can see that the variances monotonically decrease as *N* becomes larger.

Note that if the λ-condition (Equation 33) does not have a non-trivial solution like Equation (39), the expression for the PGF (Equation 42) cannot be applied. Such special cases are treated in the following section.

##### 2.4.2.4. Ergodicity as a sufficient condition for applying our PGF

The PGFwoWTAS (Equation 42) would be applicable if the catalytic reaction network was “entirely ergodic,” which means the following in this paper (it is reminiscent of the ω-limit set):

(48) $$\begin{array}{l} \omega \left(n_{0};\xi \right)=W\;\backslash \;I\;\left(\forall \xi \right),\; \end{array}$$

where

(49) $$\begin{array}{l} \omega \left(n_{0};\xi \right):=\underset{t\geq 0}{⋂}\left\{n\left(\tau ;\xi \right):\tau \geq t\right\},\; \end{array}$$

and ξ represents one of the trials of the stochastic process ***n***(*t*) according to the catalytic reaction network.

**Figure 4:**
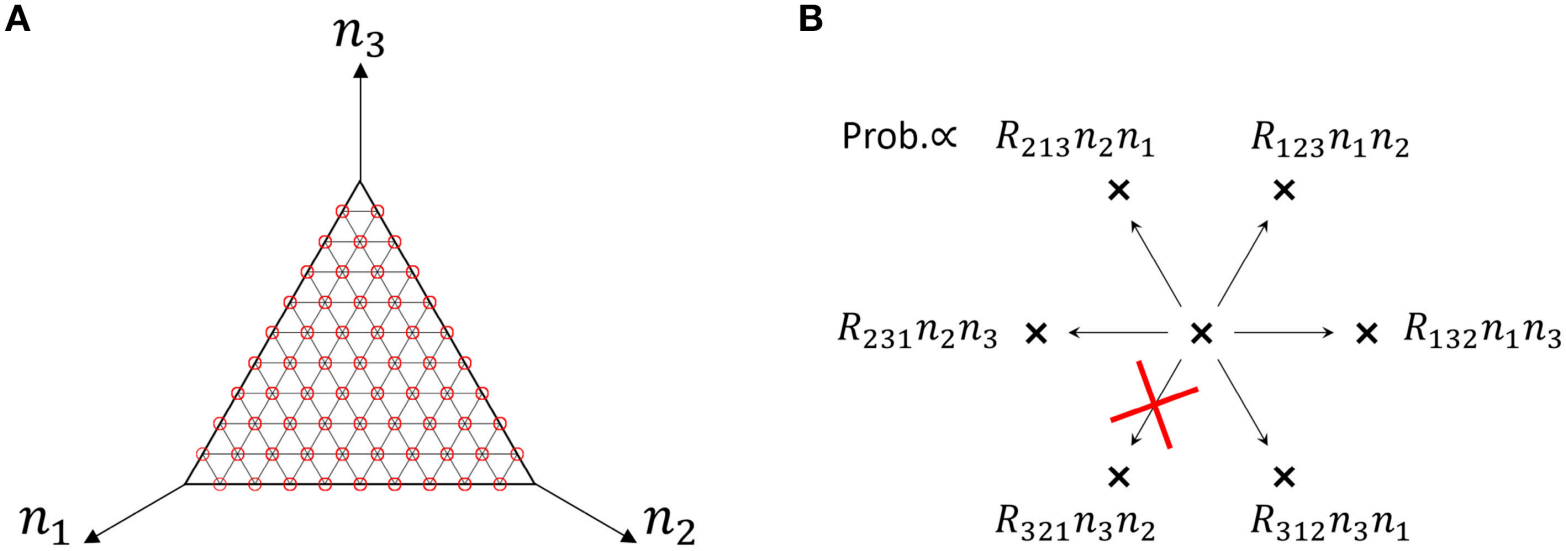
**(A)** State space of the three-species system of Figure 1A in the case of *N* = 10. Circles (red) represent the states that a single trajectory can visit, which means that the system is “entirely ergodic.” **(B)** Motion of the state point when one reaction occurs in the three-species system of Figure 1A. There are 6 possible directions in which to move from one state point. Each direction is randomly selected in proportion to the specified probability. In the case of Figure 1A, the state point cannot move in the direction of *R*_321_ since *R*_321_ = 0. Note that the state point on the boundary (e.g., *n*_1_ = 0) cannot move parallel to the boundary (in this case, the directions of *R*_213_ and *R*_312_).

**Figure 5:**
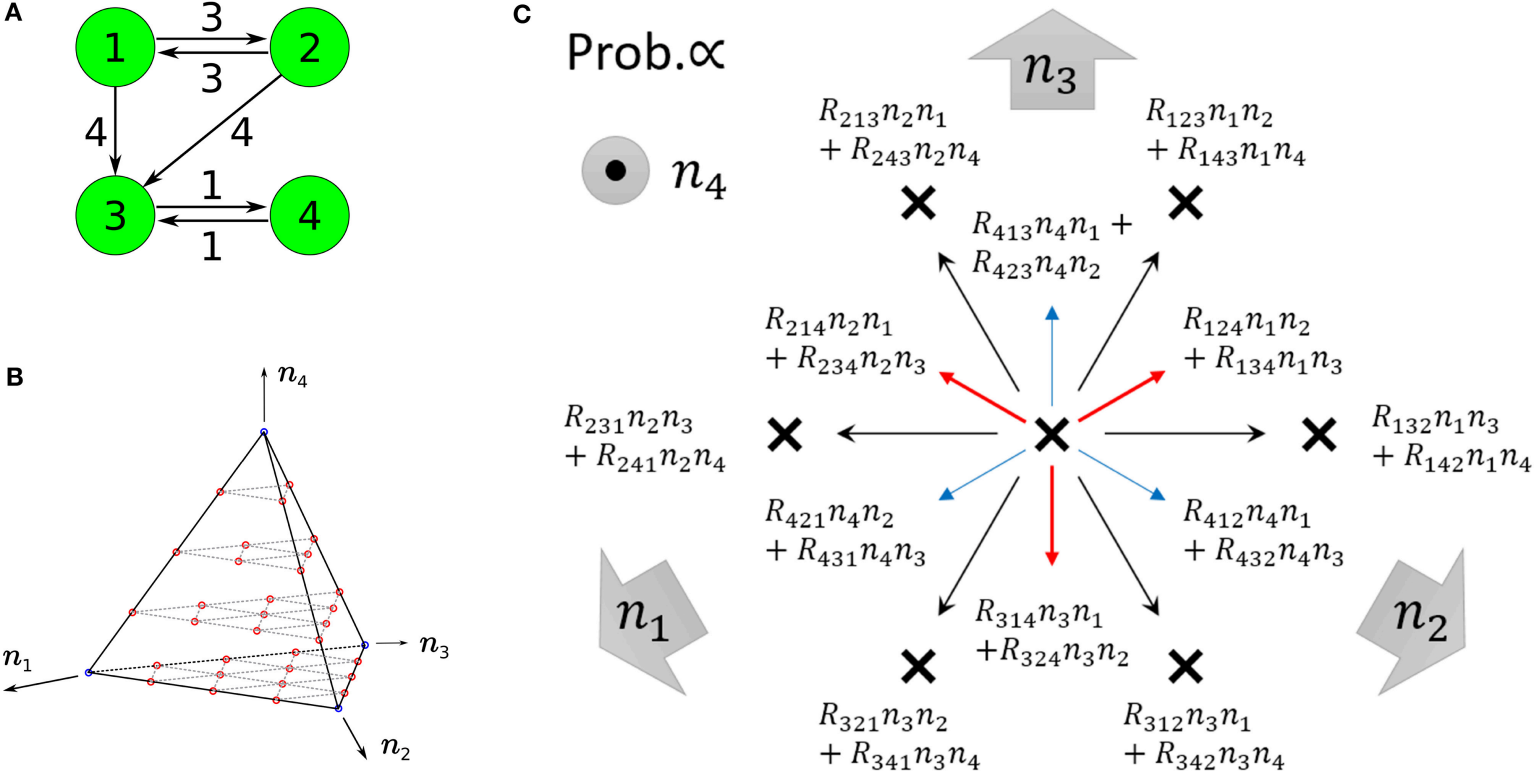
**(A)** Example of non-ergodic catalytic networks consisting of four species; the rate constants are *R*_132_ > 0, *R*_143_ > 0, *R*_231_ > 0, *R*_243_ > 0,*R*_314_ > 0, *R*_413_ > 0, and others 0. **(B)** State space of four-species systems in the case of *N* = 4. Red circles represent the states a single trajectory can visit in the case that the system is entirely ergodic. Blue circles represent winner-takes-all states, i.e., *n*_*k*_ = 4 (∃*k*). **(C)** Motion of the state point when one reaction occurs in four-species systems (viewed from the top of **B**). Red and blue arrows represent upward and downward arrows, respectively. There are 12 possible directions in which to move from one state point. Each direction is randomly selected in proportion to the specified probability. Note that the state point on each $I^{\left(2\right)}\left(i_{1},i_{2}\right)$ cannot move parallel to itself (e.g., on *I*^(2)^(1, 2), the directions of *R*_231_, *R*_241_ as well as *R*_132_, *R*_142_ are not allowed).

We use a specific three-species system to intuitively illustrate what Equation (48) means. In the case of the three-species system of Figure 1A, the ergodic condition (Equation 48) can be rewritten in terms of simplified conditions. As previously described, the state space of catalytic reaction networks consists of $\left(\left(\frac{M}{N}\right)\right):=\frac{(N+M-1)!}{(M-1)!N!}$ points. Therefore, in the case of *M* = 3 for example, there are (*N* + 2)!/(2*N*!) points. The state space of the three-species system forms a regular triangle in three-dimensional Euclidean space (see Figure 4A). The possible motion from each state point is shown in Figure 4B, where there potentially exist six possible directions, but the network structure forbids the direction of *R*_321_ in the case of the network of Figure 1A. Note that the state point on the boundary cannot move in a direction parallel to the boundary. In this case obviously, a trajectory starting from an initial point ***n***_0_ ∈ *W* \ *I* visits every point in *W* \ *I* as shown by the circles in Figure 4A (the set *I* is equivalent to the vertexes of a triangle). Therefore, as seen in Figure 4B, the necessary and sufficient condition to hold the ergodic condition (Equation 48) for *M* = 3 is:

(50) $$\begin{array}{l} \underset{i,j}{\sum }R_{ijk}>0\;\&\;\underset{i,j}{\sum }R_{kij}>0\;\left(\forall k\right),\; \end{array}$$

where the first condition represents that at least one direction for moving away from the boundary *n*_*k*_ = 0 is allowed, and the second condition represents that at least one direction for approaching the boundary *n*_*k*_ = 0 is allowed. Note that the condition (Equation 50) is no longer a sufficient condition for entire ergodicity in the case of four-species systems. In fact, in the following four-species system (Figure 5A), the condition (Equation 50) does not imply entire ergodicity;

(51) $$\begin{array}{l} R_{132}>0,\;R_{143}>0,\;R_{231}>0,\;R_{243}>0,\;R_{314}>0,\;\\ \;R_{413}>0,\;\left(\text{others }0\right).\; \end{array}$$

This four-species system obviously satisfies the condition (Equation 50), but most initial states [in particular, *n*_*k*_(0) > 0(∀*k*)] eventually fall into any of 2-species winners-take-all states, i.e., {*n*_1_ = *n*_2_ = 0, *n*_3_ + *n*_4_ = *N*}.

In the case of *M*-species systems, a necessary and sufficient condition for entire ergodicity is difficult to derive, although we may consider a sufficient condition instead. We discuss the sufficient condition for entire ergodicity by introducing the collection of all *l-species winners-take-all states* (*l* = 1, 2, ⋯ , *M*):

(52) $$\begin{array}{l} I_{M,N}^{\left(l\right)}:=\underset{1\leq i_{1}<\cdots <i_{l}\leq M}{⋃}I_{M,N}^{\left(l\right)}\left(i_{1},i_{2},\cdots \;,i_{l}\right),\; \end{array}$$

where

(53) $$\begin{array}{l} I_{M,N}^{\left(l\right)}(i_{1},i_{2},\cdots ,i_{l}):\;=\{n\in W_{M,N}|n_{i_{1}}+n_{i_{2}}+\cdots +n_{i_{l}}=N\\ \;\&\;n_{i_{k}}>0(\forall k)\}. \end{array}$$

We omit the subscripts *M* and *N* provided there is no concern for confusion. The above collections are not empty sets if *l* ≤ *N*. Note that *I* = *I*^(1)^ and $W={⋃}_{l=1}^{M}I^{\left(l\right)}$. One can see that the following diagram holds if the catalytic network is fully connected [i.e., *R*_*ijk*_ > 0 for all *i, j, k* (*i* ≠ *j* ≠ *k* ≠ *i*)];

(54) $$\begin{array}{c} I^{\left(1\right)}⇆̸I^{\left(2\right)}⇆I^{\left(3\right)}⇆\cdots ⇆I^{\left(M-1\right)}⇆I^{\left(M\right)}\\ \;↺\;↺\;↺\;↺\; \end{array}\;$$

where each arrow represents the direction in which a state point can move by one reaction. Each $I^{\left(l\right)}\left(i_{1},\cdots \;,i_{l}\right)$ is a (*l* − 1)-dimensional object in the whole (*M* − 1)-dimensional state space *W*. For example, the state space of four-species systems forms a regular tetrahedron *W*_*M* = 4, *N*_ (see Figure 5B); *I*^(4)^(1, 2, 3, 4) is the interior of the regular tetrahedron; *I*^(3)^(1, 2, 3), *I*^(3)^(1, 2, 4), *I*^(3)^(1, 3, 4), and *I*^(3)^(2, 3, 4) are regular triangles that form the boundaries of *I*^(4)^; and *I*^(2)^(1, 2), *I*^(2)^(1, 3), *I*^(2)^(1, 4), *I*^(2)^(2, 3), *I*^(2)^(2, 4), and *I*^(2)^(3, 4) are line segments that form the boundaries of *I*^(3)^. Note that $I^{\left(2\right)}\left(i_{1},i_{2}\right)\to I^{\left(2\right)}\left(j_{1},j_{2}\right)$ for (*i*_1_, *i*_2_) ≠ (*j*_1_, *j*_2_) is possible but $I^{\left(2\right)}\left(i_{1},i_{2}\right)\to I^{\left(2\right)}\left(i_{1},i_{2}\right)$ is always impossible as seen from Figure 5C. By considering the diagram (Equation 54) and keeping in mind Figures 5B,C, we can expect the following fact: *if entire ergodicity holds in the restricted system on I*^(*l*)^ *for l* ≥ 3*, then entire ergodicity holds in the whole state space W*. In other words, in systems consisting of *M*-species, the sufficient condition under which the ergodic condition (Equation 48) holds can be geometrically described as follows: *on each* $I^{\left(l\right)}\left(i_{1},\cdots \;,i_{l}\right)$ *for l* ≥ 3*, at least one direction for moving away from each boundary* $I^{\left(l-1\right)}\left(j_{1},\cdots \;,j_{l-1}\right)$ *should be allowed, and simultaneously at least one direction for approaching each boundary* $I^{\left(l-1\right)}\left(j_{1},\cdots \;,j_{l-1}\right)$ *should also be allowed, where* {*j*_1_, ⋯ , *j*_*l*−1_}⊂{*i*_1_, ⋯ , *i*_*l*_}.

#### 2.4.3. The case of catalytic-noncatalytic mixed reactions

We could not obtain the steady-state solution of the general GFE (Equation 11) in the case of catalytic-noncatalytic mixed reactions (ε > 0), but we expect our PGF (Equation 42) to be a good approximation for mixed-reaction systems if ε is sufficiently small (ε ≪ min{*R*_*ijk*_ > 0}). More specifically, we expect the PGF (Equation 42) to be robust against non-catalytic reactions if the catalytic reaction system constituting the mixed reaction system has an ergodic component spread across the entire state space (*entire ergodicity*). Otherwise, if the catalytic reaction system has several ergodic components in the entire state space, the non-catalytic reactions may imply that a certain highly stable ergodic component attracts all possible trajectories, which obviously means that our PGF is not applicable to such a mixed reaction system. Although we were unable to prove this mathematically, we used numerical simulations to determine whether our PGF is a good approximation in the case of “entirely ergodic.”

**Figure 6:**
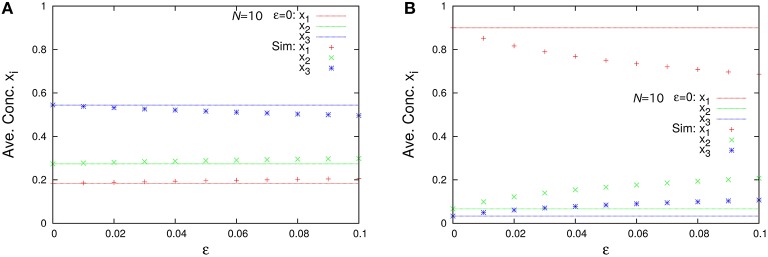
**ε-dependence of the time-averaged concentrations ***x***_***i***_ (***N*** = 10, ρ = 1) in the case of (A) the three-species system of Figure 1A with non-catalytic reactions and (B) that of Figure 1B with non-catalytic reactions**. Points in the figures are numerically obtained using the Gillespie algorithm in the same way as Figure 3. The catalytic reaction network of Figure 1A is entirely ergodic; thus, the non-catalytic reactions may be treated as perturbation. On the other hand, that of Figure 1B is non-ergodic; thus, the non-catalytic reactions introduce relatively large differences compared with the case of ε = 0.

Figure 6 represents the non-catalytic reaction rate constant ε dependencies of time-averaged concentrations *x*_*i*_ for the three-species system of Figures 1A,B. (As shown later, the system shown in Figure 1B is not entirely ergodic, which is the reason why the systems shown in Figures 1A,B are compared here.) It can be seen that the differences of *x*_*i*_ in Figure 6A between ε = 0 and ε = 0.1 are smaller than those in Figure 6B. We consider the robustness in the case of Figure 6A to originate from the entire ergodicity of Figure 1A, which means that the ergodic component spreads on the entire state space except for winner-takes-all states (see Figure 4A).

## 3. Applications

The starting point of our analysis is the GFE (Equation 11), from which several useful formulas are derived, namely the PRE (Equation 15), SME (Equation 18), TESMs (Equation 19) and (Equation 20), the λ-condition (Equation 33), and the PGFwoWTAS (Equation 42). In this section, we reveal the effectiveness of these formulas by showing important applications for several catalytic reaction networks.

### 3.1. Rank conservation law for concentrations

We show that the rank of concentrations is conserved even if the total number of molecules changes in catalytic reaction networks (excluding non-catalytic and auto-catalytic reactions).

Suppose the concentration of the *i*th-species is expressed by Equation (45a) in the state corresponding to the PGFwoWTAS. When determining the rank conservation, it suffices to confirm that the relation between the amount of two arbitrary species is unchanged if the total number of molecules is changed. Let λ_1_, λ_2_ be the concentrations per total density in the continuous limit such that λ_1_ < λ_2_. Because $\overset{M}{\underset{i=1}{\sum }}\lambda_{i}=1$, the inequality λ_1_ + λ_2_ < 1 must be satisfied. Therefore, the following evaluation holds:

(55) $$\begin{array}{l} {\bar{x}}_{2}-{\bar{x}}_{1}=\frac{\left(\lambda_{2}-\lambda_{2}^{N})-(\lambda_{1}-\lambda_{1}^{N}\right)}{1-\sum_{i=1}^{M}\lambda_{i}^{N}}\rho \\ \;=\frac{(\lambda_{2}-\lambda_{1})\rho }{1-\sum_{i=1}^{M}\lambda_{i}^{N}}[1-{\left(\lambda_{2}+\lambda_{1}\right)}^{N-1}\\ \;\;+\sum_{k=1}^{N-2}\left\{\left(\begin{array}{c} N-1\\ k \end{array}\right)-1\right\}\lambda_{2}^{N-1-k}\lambda_{1}^{k}]\\ \;>0\;(\forall N\in {\mathbb{N}}_{\geq 2}). \end{array}$$

This is the rank conservation law of concentrations.

Note that the rank of the variances of concentrations is generally not conserved when the total number of molecules changes. For example, let us consider the following three-species system (Figure 1C);

(56) $$\begin{array}{l} R_{123}=1997/3,\;R_{132}=1000/3,\;R_{231}=R_{321}=1,\;\\ \;R_{213}=R_{312}=0;\;\rho =1.\; \end{array}$$

**Figure 7:**
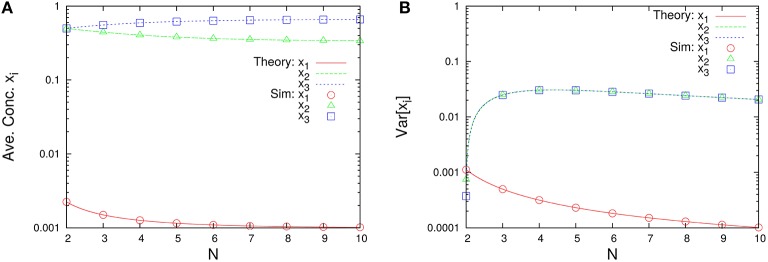
*****N***-dependence of (A) the time-averaged concentrations ${\bar{x}}_{i}={\bar{n}}_{i}/N$ and (B) the variances of concentrations $\text{Var}\left[x_{i}\right]=\left(\bar{n_{i}^{2}}-{\bar{n}}_{i}^{2}\right)/N^{2}$ in the three-species system of Figure 1C**. Empty symbols are numerically obtained using the Gillespie algorithm in the same way as Figure 3. Lines in each figure represent the theoretical expressions, Equations (45a) and (45c), for λ_1_ = 1/1000, λ_2_ = 1/3, and λ_3_ = 1997/3000. It is clear that the rank of time-averaged concentrations is conserved.

**Figure 8:**
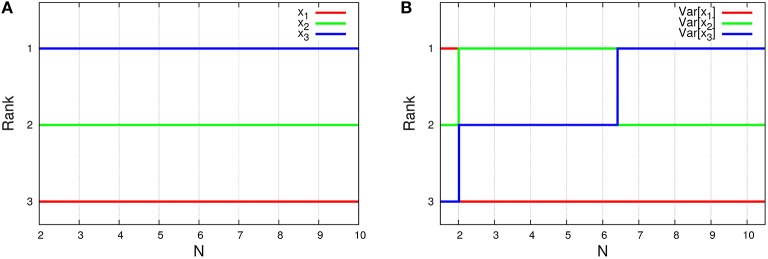
*****N***-dependence of (A) the rank of time-averaged concentrations and (B) the rank of its variances in the three-species system of Figure 1C**. The ranks (lines) in the figures are depicted using the formulas (Equations 45a and 45c). Clearly, the rank of concentrations is conserved, although the rank of variances is exchanged between *N* = 2 and 3, and also *N* = 6 and 7.

As shown in Figure 7, this system depends on the total number of molecules for the time-averaged concentrations ${\bar{x}}_{i}$ and the variances of concentrations Var[*x*_*i*_], represented by Equations (45a) and (45c) with λ_1_ = 1/1000, λ_2_ = 1/3, and λ_3_ = 1997/3000. The rank of time-averaged concentrations is always conserved, but the rank of variances is exchanged at certain *N* (see Figure 8).

### 3.2. A special case: the connecting state to the winner-takes-all state

There exists an *N*-molecular state, which connects to the winner-takes-all state in the continuous limit *N* → ∞. As is later shown, such a special case is not the “weakly reversible” case (Anderson et al., 2010).

We show this by taking the following limit in the PGFwoWTAS (Equation 42):

(57) $$\begin{array}{l} \lambda_{1}\to 1,\;\lambda_{2},\cdots \;,\lambda_{M}\to 0,\; \end{array}$$

where we suppose the following constraints are always satisfied:

(58) $$\begin{array}{l} \frac{\lambda_{i}}{\lambda_{2}+\lambda_{3}+\cdots +\lambda_{M}}=\kappa_{i}\;\left(i=2,3,\cdots \;,M\right),\; \end{array}$$

where {κ_*i*_} are positive constants, which are determined from the network structure {*R*_*ijk*_} (it is explained later). Evidently, the following holds;

(59) $$\begin{array}{l} \overset{M}{\underset{i=2}{\sum }}\kappa_{i}=1.\; \end{array}$$

The PGFwoWTAS (Equation 42) has the following limiting expression:

(60) $$\begin{array}{l} \phi_{*}^{\text{c}}\left(z\right)=\frac{{\left(\frac{\lambda_{1}}{1-\lambda_{1}}z_{1}+\sum_{i=2}^{M}\kappa_{i}z_{i}\right)}^{N}-{\left(\frac{\lambda_{1}}{1-\lambda_{1}}z_{1}\right)}^{N}-\sum_{i=2}^{M}{\left(\kappa_{i}z_{i}\right)}^{N}}{{\left(\frac{1}{1-\lambda_{1}}\right)}^{N}-{\left(\frac{\lambda_{1}}{1-\lambda_{1}}\right)}^{N}-\sum_{i=2}^{M}\kappa_{i}^{N}},\;\\ \overset{}{\underset{\lambda_{1}\to 1}{\to }}\;z_{1}^{N-1}\left(\kappa_{2}z_{2}+\cdots +\kappa_{M}z_{M}\right)\equiv \phi_{*}^{\text{cs}}\left(z\right).\; \end{array}$$

The state corresponding to the PGF (Equation 60) is the connecting state to the winner-takes-all state (CStoWTAS). The stationary distribution corresponding to the CStoWTAS is immediately obtained:

(61) $$P_{*}^{\text{cs}}(n)=\{\begin{array}{c} \kappa_{i}\;\;\;\;\;(n\in J),\\ 0\;\;\;\;\;(n\in W\backslash J), \end{array}$$

where *J*_*M, N*_ (abbr. *J*) is defined as

(62) $$\begin{array}{l} J=\left\{n\in W_{M,N}|n_{1}=N-1\;\&\;\exists i\in \left[2,M\right]\text{ s.t. }n_{i}=1\right\}.\; \end{array}$$

Furthermore, the marginal distributions of the *i*-th species can be derived by Equation (13):

(63) $$\begin{array}{l} p_{i\;*}^{\text{cs}}(n)=\{\begin{array}{ll} \delta_{n,N-1} & \text{for }i=1,\\ (1-\kappa_{i})\delta_{n,0}+\kappa_{i}\delta_{n,1} & \text{for }i=2,\cdots ,M, \end{array}\\ \;(0\leq n\leq N), \end{array}$$

where δ_*ij*_ is the Kronecker delta, δ_*ij*_ = 0 (if *i* ≠ *j*) or 1 (if *i* = *j*). The time-averaged concentrations calculated by Equation (12) are

(64a) $$\begin{array}{l} {\bar{x}}_{1}=\left(1-\frac{1}{N}\right)\;\rho ,\; \end{array}$$

(64b) $$\begin{array}{l} {\bar{x}}_{i}=\frac{\kappa_{i}}{N}\rho \;\left(i=2,\cdots \;,M\right),\; \end{array}$$

and the variances of the time-averaged concentrations calculated by Equation (12) are

(65a) $$\begin{array}{l} \text{Var}\left[x_{1}\right]=0,\; \end{array}$$

(65b) $$\begin{array}{l} \text{Var}\left[x_{i}\right]=\frac{\kappa_{i}\left(1-\kappa_{i}\right)}{N^{2}}\rho^{2}\;\left(i=2,\cdots \;,M\right).\; \end{array}$$

Next, we derive the relation between the positive constants {κ_*i*_} and the network structure {*R*_*ijk*_}. The λ-condition (Equation 33) can be converged to conditions for {κ_*i*_} (the κ-condition) by taking the limit λ_1_ → 1 as follows. Divide the λ-condition (Equation 33) into two groups, of which one group is the case of 2 ≤ *i* < *j* ≤ *M*,

(66a) $$\begin{array}{l} R_{1ij}\lambda_{1}\kappa_{i}+R_{1ji}\lambda_{1}\kappa_{j}-\left(R_{ij1}+R_{ji1}\right)\kappa_{i}\kappa_{j}\left(1-\lambda_{1}\right)\;\\ +\left(1-\lambda_{1}\right)\overset{M}{\underset{k=2}{\sum }}\left\{R_{kij}\kappa_{k}\kappa_{i}+R_{kji}\kappa_{k}\kappa_{j}-\left(R_{ijk}+R_{jik}\right)\kappa_{i}\kappa_{j}\right\}=0,\; \end{array}$$

and the other group is the case of *i* = 1, 2 ≤ *j* ≤ *M*,

(66b) $$\begin{array}{l} \overset{M}{\underset{k=2}{\sum }}\left\{R_{k1j}\kappa_{k}\lambda_{1}+R_{kj1}\kappa_{k}\kappa_{j}\left(1-\lambda_{1}\right)-\left(R_{1jk}+R_{j1k}\right)\lambda_{1}\kappa_{j}\right\}=0,\; \end{array}$$

where we substituted λ_*i*_ = κ_*i*_(1− λ _1_), *i* ≥ 2 and divided by 1− λ _1_. Then, taking the limit λ_1_ → 1 in Equation (66a),

(67a) $$\begin{array}{l} \kappa_{i}R_{1ij}+\kappa_{j}R_{1ji}=0\text{ i.e., }R_{1ij}=0\;\left(\forall i,j\right),\; \end{array}$$

and taking that in Equation (66b),

(67b) $$\begin{array}{l} \overset{M}{\underset{k=2}{\sum }}\left(\kappa_{k}R_{k1j}-\kappa_{j}R_{j1k}\right)=0\;\left(2\leq j\leq M\right).\; \end{array}$$

The condition (Equation 67a) expresses that the 1st-species cannot be a substrate, and the condition (Equation 67b) represents the desired κ-condition. Note that the CStoWTAS must be a limiting state corresponding to the PGFwoWTAS [see Equation (60)]. Therefore, the network structure {*R*_*ijk*_} must have definite {λ_*i*_}.

**Figure 9:**
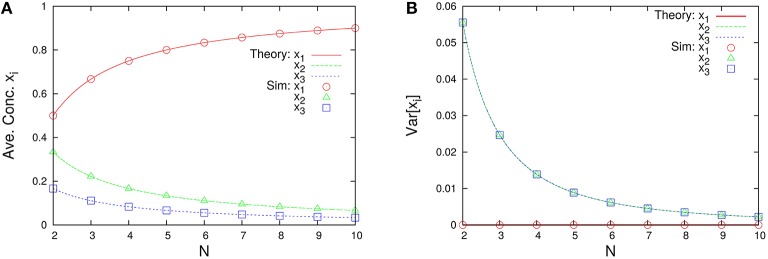
*****N***-dependence of (A) time-averaged concentrations ${\bar{x}}_{i}={\bar{n}}_{i}/N$ and (B) the variances of concentrations $\text{Var}\left[x_{i}\right]=\left(\bar{n_{i}^{2}}-{\bar{n}}_{i}^{2}\right)/N^{2}$ in the three-species system of Figure 1B**. Empty symbols were numerically obtained using the Gillespie algorithm in the same way as Figure 3. Lines in each figure represent the theoretical expressions, Equations (64) and (65), for κ_2_ = 2/3 and κ_3_ = 1/3.

For example, let us consider the following three-species system (Figure 1B):

(68) $$\begin{array}{l} R_{123}=R_{132}=0,R_{213}=R_{231}=R_{321}=1,R_{312}=2;\rho =1,\; \end{array}$$

which implies κ_2_ = 2/3 and κ_3_ = 1/3. Obviously, this system is not weakly reversible, which means that the theorems (Theorem 4.1 and 4.2) in the previous study (Anderson et al., 2010) cannot be applied. This system should have a CStoWTAS because λ_1_, λ_2_, and λ_3_ are definite from Equation (39) with Λ_1_ = 0, Λ_2_ = 2, and Λ_3_ = 4. As shown in Figure 9, this system certainly has the time-averaged concentrations ${\bar{x}}_{i}$ represented by Equation (64) and the variances *Var*[*x*_*i*_] represented by Equation (65). In this case also, the rank conservation law of concentrations holds.

### 3.3. *M*-species 2-molecules systems with non-catalytic reactions

The 2mTESM (Equation 20) becomes the closed equation of the second-order moments 〈*x*_*l*_*x*_*m*_〉 if the first-order moments 〈*x*_*i*_〉 are substituted by the second-order moments according to the PRE (Equation 20). In this subsection, we consider catalytic-non-catalytic mixed reaction systems of *M* species, consisting of only 2 molecules in total.

We first focus on the second formula in the 2mTESMs (Equation 20). It can be seen that each variance of time-averaged concentration $\text{Var}\left[x_{i}\right]=\bar{x_{i}^{2}}-{\bar{x_{i}}}^{2}$ in the steady state depends solely on its concentration:

(69) $$\begin{array}{l} \text{Var}\left[x_{i}\right]=\left(\frac{M+1}{2M}\rho -{\bar{x}}_{i}\right){\bar{x}}_{i},\; \end{array}$$

where we supposed the ergodicity $\langle x_{i}\rangle ={\bar{x}}_{i}$. The above equation expresses that the fluctuation of the concentration *x*_*i*_ becomes larger as its time-averaged concentration approaches

(70) $$\begin{array}{l} \bar{x}=\frac{M+1}{4M}\rho ,\; \end{array}$$

at which the fluctuation takes the largest value ${\left(\frac{M+1}{4M}\rho \right)}^{2}$. Furthermore, the time-averaged concentrations potentially have the maximum value because the variance must be positive;

(71) $$\begin{array}{l} {\bar{x}}_{i}\leq \frac{M+1}{2M}\rho \;\left(\forall i\in \left[1,M\right]\right).\; \end{array}$$

Next, we focus on the first formula in the 2mTESMs (Equation 20). Let us consider the steady state of Equation (20a) and suppose the ergodicity $\langle x_{i}\rangle ={\bar{x}}_{i}$. Eliminating the first-order moments in Equation (20a) by using the SME (Equation 18) gives the determination equation of second-order moments in the 2 molecules system (2mDESM);

(72) $$\begin{array}{l} \frac{4\varepsilon }{\rho }\bar{x_{l}x_{m}}+\underset{i}{\sum }\left\{\left(R_{lmi}+R_{mli}\right)\bar{x_{l}x_{m}}-R_{ilm}\bar{x_{i}x_{l}}-R_{iml}\bar{x_{i}x_{m}}\right\}\;\\ \;-\frac{1}{M}\underset{ij}{\sum }\left\{\left(R_{jil}+R_{jim}\right)\bar{x_{i}x_{j}}-R_{lij}\bar{x_{i}x_{l}}-R_{mij}\bar{x_{i}x_{m}}\right\}\;\\ \;=\frac{2\rho \varepsilon }{M^{2}}\;\left(1\leq l<m\leq M\right).\; \end{array}$$

The above 2mDESM can be rewritten in the *M*(*M*−1)/2 × *M*(*M*−1)/2 matrix form. We demonstrate the procedure for processing the 2mDESM by considering the case of *M* = 3. In this case, the 2mDESM (Equation 72) has a matrix of the following form:

(73) $$\begin{array}{l} \left[\begin{array}{ccc} \frac{4}{3}(R_{123}+R_{213})+\text{​}\frac{4\varepsilon }{\rho } & \text{​}-\frac{4}{3}R_{312} & \text{​}-\frac{4}{3}R_{321}\\ -\frac{4}{3}R_{213} & \text{​}\frac{4}{3}(R_{132}+R_{312})+\text{​}\frac{4\varepsilon }{\rho } & \text{​}-\frac{4}{3}R_{231}\\ -\frac{4}{3}R_{123} & \text{​}-\frac{4}{3}R_{132} & \text{​}\frac{4}{3}(R_{231}+R_{321})+\text{​}\frac{4\varepsilon }{\rho } \end{array}\right]\\ \;\left[\begin{array}{c} \bar{x_{1}x_{2}}\\ \bar{x_{1}x_{3}}\\ \bar{x_{2}x_{3}} \end{array}\right]=\frac{2\rho \varepsilon }{9}\left[\begin{array}{c} 1\\ 1\\ 1 \end{array}\right]. \end{array}$$

Moreover, we consider the specific three-species system of Figure 1A (ρ = 1) including non-catalytic reactions. Solving Equation (73) as ε > 0 and ρ = 1 in the case of Figure 1A, then

(74) $$\left[\begin{array}{c} \bar{x_{1}x_{2}}\\ \bar{x_{1}x_{3}}\\ \bar{x_{2}x_{3}} \end{array}\right]=\left[\begin{array}{c} \frac{1+3\varepsilon }{18(2+3\varepsilon )}\\ \frac{1}{18}\\ \frac{1+\varepsilon }{6(2+3\varepsilon )} \end{array}\right],$$

which is consistent with the result obtained when solving the CME (Equation 7) directly [recall $\langle x_{1}x_{2}\rangle =\frac{1}{4}P\left(n_{1}=1,n_{2}=1,n_{3}=0\right)$, 〈*x*_1_*x*_3_〉 = ⋯  and so on]. By the SME (Equation 18), the concentrations become

(75) $$\begin{array}{l} {\bar{x}}_{1}=\frac{1+2\varepsilon }{2\left(2+3\varepsilon \right)},\;{\bar{x}}_{2}=\frac{1}{3},\;{\bar{x}}_{3}=\frac{5+6\varepsilon }{6\left(2+3\varepsilon \right)}.\; \end{array}$$

**Figure 10:**
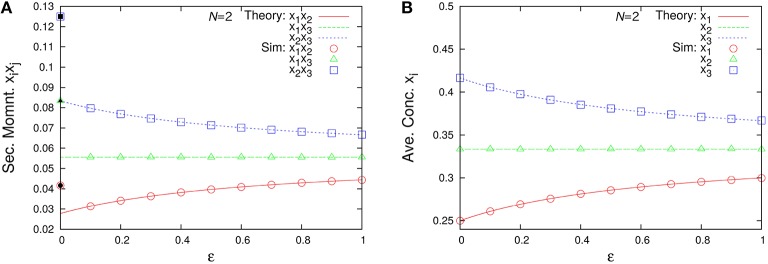
**ε-dependence of (A) the second-order moments $\bar{x_{i}x_{j}}=\bar{n_{i}n_{j}}/N^{2}$ and (B) the time-averaged concentrations ${\bar{x}}_{i}={\bar{n}}_{i}/N$ in the three-species system of Figure 1A with non-catalytic reactions (***N*** = 2, ρ = 1)**. Empty symbols are numerically obtained using the Gillespie algorithm in the same way as Figure 3. Lines in each figure represent the theoretical expressions, Equations (74) and (75). Note that there exist differences between the limit ε → 0 and the case ε = 0 for the second-order moments (solid black points) because the SME (Equation 18) breaks down at ε = 0. The solid black points are calculated from Equation (45b) as λ_1_ = 2/11, λ_2_ = 3/11, λ_3_ = 6/11, *N* = 2, and ρ = 1.

Figure 10 shows the second-order moments $\bar{x_{i}x_{j}}$ and the time-averaged concentrations ${\bar{x}}_{i}$ as functions of ε. Note that in the case of ε = 0 (catalytic reactions only), we need to derive the second-order moments $\bar{x_{i}x_{j}}$ from Equation (45b) corresponding to λ_1_ = 2/11, λ_2_ = 3/11, and λ_3_ = 6/11. The non-catalytic reaction rate constant ε seems to be a singular perturbation against the second-order moments (not the concentrations).

### 3.4. Non-autocatalyzation of autocatalytic reaction networks

The framework we developed in this paper applies to non-autocatalytic reaction networks. However, our framework may be applicable to autocatalytic reaction networks if it were possible to convert autocatalytic to non-autocatalytic networks. Here, we show several examples of such conversions using a minimal autocatalytic reaction network “2TK model” (Ohkubo et al., 2008; Saito and Kaneko, 2015), which is a well-studied model in the context of discreteness-induced phenomena.

**Figure 11:**
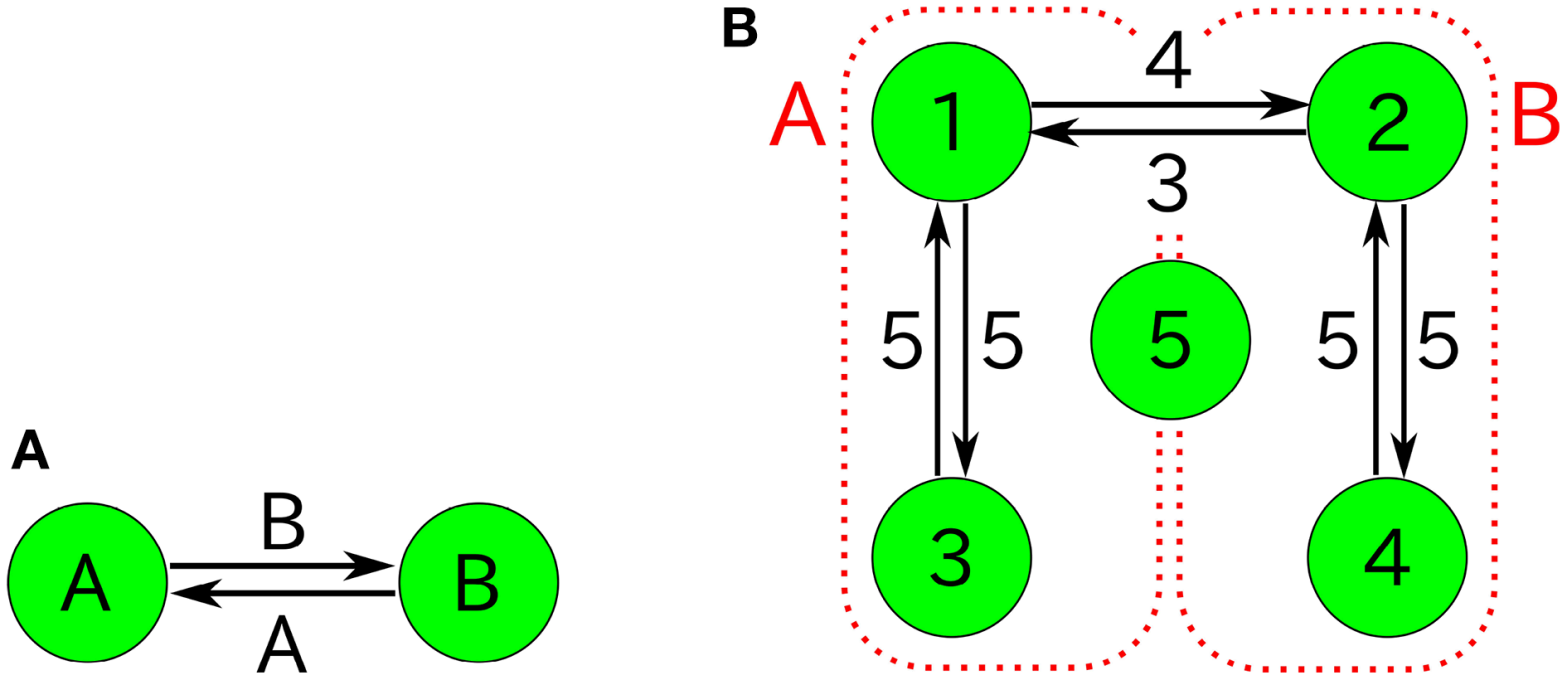
**(A)** Autocatalytic reactions constituting the 2TK model and **(B)** five-component non-autocatalytic reactions duplicating the behavior of the 2TK model; *R*_142_ = *R*_153_ = *R*_231_ = *R*_254_ = *R*_351_ = *R*_452_ = 1 (others 0). If one regards the species 1, 3, and half of 5 as the species *A* (similarly, 2, 4, and half 5 as *B*), the behavior of *n*_*A*_ = *n*_1_+*n*_3_+*n*_5_/2 and *n*_*B*_ = *n*_2_+*n*_4_+*n*_5_/2 is similar to that of the 2TK model.

The 2TK model consists of only two species, and includes both autocatalytic reactions (rate const. *r*) and non-catalytic reactions (rate const. ε ≪ *r*) (see Figure 11A);

(76) $$\begin{array}{l} A\overset{B}{\underset{A}{⇄}}B\;\left(\text{autocat. react.}\right),\; \end{array}$$

(77) $$\begin{array}{l} A⇄B\;\left(\text{non-cat. react.}\right).\; \end{array}$$

If we suppose that each of the species *A* and *B* consists of two further species, we can convert autocatalytic reactions Equation (76) of the 2TK model to non-autocatalytic reactions as shown in Figure 11B. The catalysis of the species 5 between the species 1 and 3 (or, 2 and 4) is to establish an equilibrium between the concentrations of the species 1 and 3 (or, 2 and 4), that would make it possible to regard these as one species. The non-autocatalytic reactions of Figure 11B plus non-catalytic reactions $i\overset{\text{Prob.}1/5}{\underset{}{\to }}j$ is the desired five-component model, of which the CME is simply described by Equation (7) with

(78) $$\begin{array}{l} R_{142}=R_{153}=R_{231}=R_{254}=R_{351}=R_{452}=1\;\left(\text{others }0\right).\; \end{array}$$

**Figure 12:**
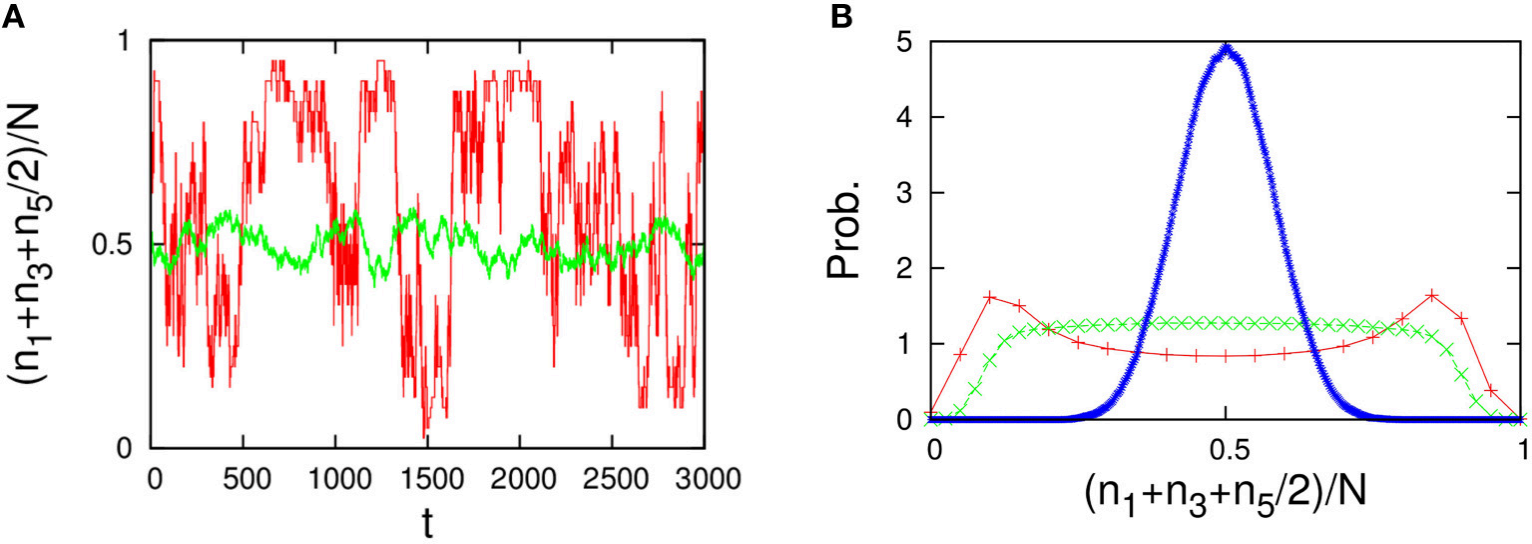
**Behavior of the five-component model [Figure 11B plus non-catalytic reactions (ε = 0.01, ρ = 1)]**. The behavior of the five-component model is reminiscent of the behavior of the 2TK model (compare with Figures 1A,B in Saito and Kaneko, 2015). **(A)** Time series of the total concentration of the species 1, 3, and half of 5 for *N* = 20 (red line) and *N* = 2000 (green line). **(B)** Stationary distributions of (*n*_1_+*n*_3_+*n*_5_/2)/*N*, obtained numerically from a long time series *n*_*i*_(*t*) using the Gillespie algorithm whose simulation conditions are the total number of reactions: 10^8^, the number of reactions for transient exclusion: 10^7^, and the initial value is randomly selected from *W* \ *I* such that the average per one-species is *N*/*M*. The unimodal distribution (blue), the flat distribution (green), and the bimodal distribution (red) indicate the stationary distribution for *N* = 500, 40, and 20, respectively.

As shown in Figure 12, the behavior of the variables *n*_*A*_ = *n*_1_+*n*_3_+*n*_5_/2 and *n*_*B*_ = *n*_2_+*n*_4_+*n*_5_/2 in the five-component model is similar to that of the 2TK model (compare with Figures 1A,B in Saito and Kaneko, 2015). The λ-condition (Equation 33) of catalytic reactions in the five-component model can be written in matrix form,

(79) $$[\begin{array}{cccccccccc} 0 & 0 & 0 & 0 & 0 & 0 & 0 & 0 & 0 & 0\\ 0 & 0 & 0 & 0 & 1 & 0 & 0 & 0 & 0 & 0\\ 0 & 0 & -1 & 0 & 0 & 0 & 0 & 0 & 0 & 0\\ 0 & 0 & 0 & -1 & 0 & 0 & 0 & 0 & 1 & 0\\ 0 & 0 & 0 & 0 & -1 & 0 & 0 & 0 & 0 & 0\\ 0 & 0 & 1 & 0 & 0 & 0 & 0 & 0 & 0 & 0\\ 0 & 0 & 0 & 0 & 0 & 0 & -1 & 0 & 0 & 1\\ 0 & 0 & 0 & 0 & 0 & 0 & 0 & 0 & 0 & 0\\ 0 & 0 & 0 & 1 & 0 & 0 & 0 & 0 & -1 & 0\\ 0 & 0 & 0 & 0 & 0 & 0 & 1 & 0 & 0 & -1 \end{array}]\;\left[\begin{array}{c} \lambda_{1}\lambda_{2}\\ \lambda_{1}\lambda_{3}\\ \lambda_{1}\lambda_{4}\\ \lambda_{1}\lambda_{5}\\ \lambda_{2}\lambda_{3}\\ \lambda_{2}\lambda_{4}\\ \lambda_{2}\lambda_{5}\\ \lambda_{3}\lambda_{4}\\ \lambda_{3}\lambda_{5}\\ \lambda_{4}\lambda_{5} \end{array}\right]=0,$$

in which there exist six non-trivial eigenvectors corresponding to the 0-eigenvalue; hence, the general eigenvector corresponding to the 0-eigenvalue can be expressed as a linear summation of those six non-trivial eigenvectors:

(80) $$\left[\begin{array}{c} \lambda_{1}\lambda_{2}\\ \lambda_{1}\lambda_{3}\\ \lambda_{1}\lambda_{4}\\ \lambda_{1}\lambda_{5}\\ \lambda_{2}\lambda_{3}\\ \lambda_{2}\lambda_{4}\\ \lambda_{2}\lambda_{5}\\ \lambda_{3}\lambda_{4}\\ \lambda_{3}\lambda_{5}\\ \lambda_{4}\lambda_{5} \end{array}\right]=c_{1}\left[\begin{array}{c} 1\\ 0\\ 0\\ 0\\ 0\\ 0\\ 0\\ 0\\ 0\\ 0 \end{array}\right]+c_{2}\left[\begin{array}{c} 0\\ 1\\ 0\\ 0\\ 0\\ 0\\ 0\\ 0\\ 0\\ 0 \end{array}\right]+c_{3}\left[\begin{array}{c} 0\\ 0\\ 0\\ 0\\ 0\\ 1\\ 0\\ 0\\ 0\\ 0 \end{array}\right]+c_{4}\left[\begin{array}{c} 0\\ 0\\ 0\\ 0\\ 0\\ 0\\ 0\\ 1\\ 0\\ 0 \end{array}\right]+c_{5}\left[\begin{array}{c} 0\\ 0\\ 0\\ 1\\ 0\\ 0\\ 0\\ 0\\ 1\\ 0 \end{array}\right]+c_{6}\left[\begin{array}{c} 0\\ 0\\ 0\\ 0\\ 0\\ 0\\ 1\\ 0\\ 0\\ 1 \end{array}\right].$$

The above equation can be used to derive six types of non-trivial solutions (λ_1_, λ_2_, ⋯ , λ_5_), which immediately correspond to the stationary states of catalytic reactions in the five-component model by using Equation (43):

(81) $$(\lambda_{1},\lambda_{2},\lambda_{3},\lambda_{4},\lambda_{5})=\{\begin{array}{ll} \text{(i)} & (0,c,0,c,1-2c),\\ \text{(ii)} & (c,0,c,0,1-2c),\\ \text{(iii)} & (2c,1-2c,0,0,0),\\ \text{(iv)} & (2c,0,1-2c,0,0),\\ \text{(v)} & (0,2c,0,1-2c,0),\\ \text{(vi)} & (0,0,2c,1-2c,0), \end{array}\;\forall c\in (0,1/2),$$

where each case (i-vi) corresponds to (i) $c_{3}=c^{2}$, *c*_6_ = *c*(1−2*c*) (others 0), (ii) $c_{2}=c^{2}$, *c*_5_ = *c*(1−2*c*) (others 0), (iii) *c*_1_ = 2*c*(1−2*c*) (others 0), (iv) *c*_2_ = 2*c*(1−2*c*) (others 0), (v) *c*_3_ = 2*c*(1−2*c*) (others 0), and (vi) *c*_4_ = 2*c*(1−2*c*) (others 0). The switching behavior of the five-component model may be explained in terms of transition processes between the above six types of steady states and the five trivial steady states λ_*i*_ = 1 (others 0) of catalytic reactions in the five-component model, which are sometimes caused by non-catalytic reactions. Furthermore, the marginal distribution of the species *A*, *p*_*A*_(*n*), can be derived by calculating the convolution of the marginal distributions *p*_1_(*n*), *p*_3_(*n*), and *p*_5_(*n*), if *p*_1_(*n*), *p*_3_(*n*), and *p*_5_(*n*) were obtained for the case including non-catalytic reactions.

**Figure 13:**
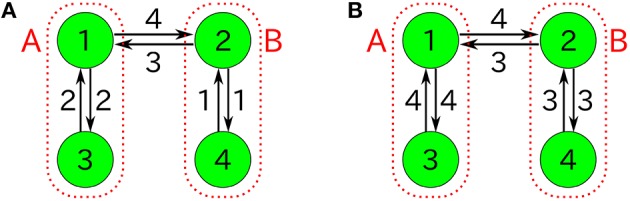
**Four-component non-autocatalytic reactions duplicating behaviors of the 2TK model; (A) ***R***_**123**_ = ***R***_**142**_ = ***R***_**214**_ = ***R***_**231**_ = ***R***_**321**_ = ***R***_**412**_ = 1 (others 0), and (B) ***R***_**142**_ = ***R***_**143**_ = ***R***_**231**_ = ***R***_**234**_ = ***R***_**341**_ = ***R***_**432**_ = 1 (others 0)**. If one regards the species 1 and 3 as the species *A* (similarly, 2 and 4 as *B*), the behavior of *n*_*A*_ = *n*_1_+*n*_3_ and *n*_*B*_ = *n*_2_+*n*_4_ is almost equivalent to that of the 2TK model.

**Figure 14:**
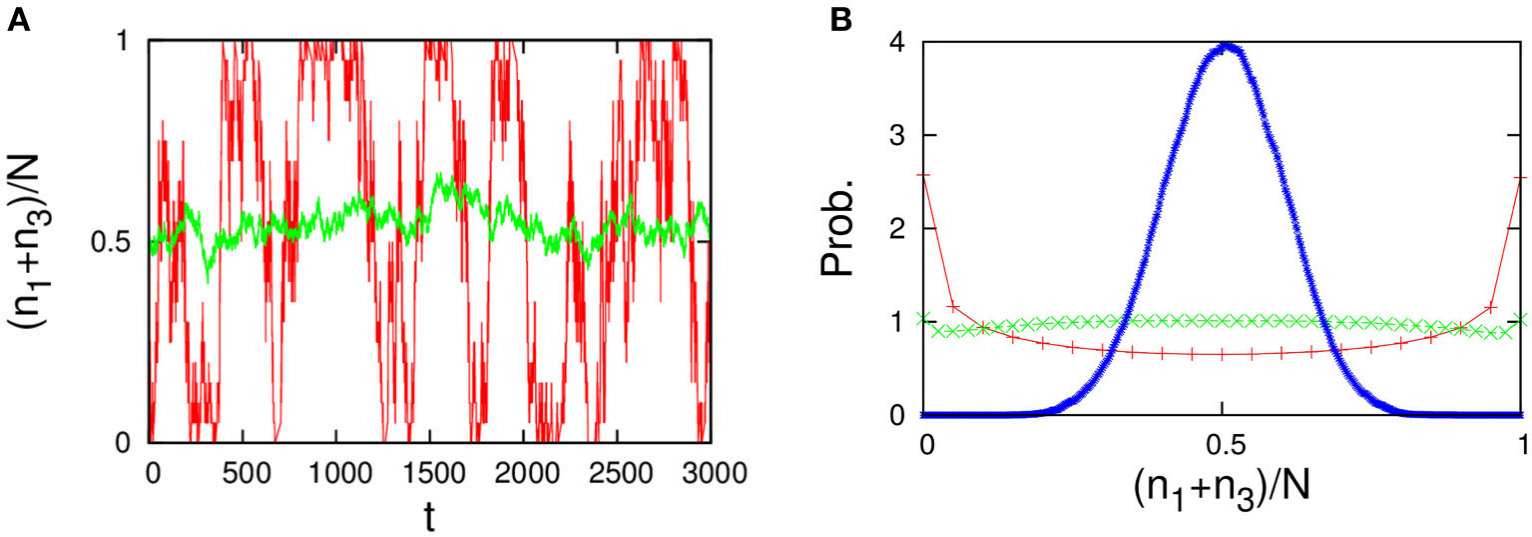
**Behavior of the four-component model [Figure 13A plus non-catalytic reactions (ε = 0.01, ρ = 1)]**. The four-component model reproduces the behavior of the 2TK model (compare with Figures 1A,B in Saito and Kaneko, 2015). **(A)** Time series of the total concentration of the species 1 and 3 for *N* = 20 (red line) and *N* = 2000 (green line). **(B)** Stationary distributions of (*n*_1_+*n*_3_)/*N*, obtained numerically from a long time series *n*_*i*_(*t*) using the Gillespie algorithm in the same way as Figure 12. The unimodal distribution (blue), the uniform distribution (green), and the bimodal distribution (red) indicate the stationary distribution for *N* = 500, 40, and 20, respectively.

Other non-autocatalytic reaction networks duplicating the 2TK model are shown in Figure 13A, and consist of 4 components only. Although readers would think that the four-component models do not function properly because the transitions between the species group (1, 3) [or (2, 4)] depend on the other species group (2, 4) [or (1, 3)], the four-component models actually generate much the same behavior with the 2TK model (see Figure 14). The switching behavior of the four-component model may also be explained as a transition process between the following three types of steady states

(82) $$(\lambda_{1},\lambda_{2},\lambda_{3},\lambda_{4})=\{\begin{array}{l} (0,0,c,1-c),\\ (0,c,0,1-c),\\ (c,0,1-c,0), \end{array}\;\forall c\in (0,1),$$

and the four trivial steady states λ_*i*_ = 1 (others 0) of catalytic reactions in the four-component model, which are sometimes caused by non-catalytic reactions. We also confirmed that the results are not changed even in the other four-component model of Figure 13B, of which the catalysis role of each the species 1 and 2 is exchanged by that of 3 and 4, respectively.

## 4. Summary and discussions

The framework we presented in this paper facilitates the prediction of the effect of the small-number issue on the concentration of each species in catalytic reaction networks. This can be described in an extreme manner by comparing the concentrations between the continuous limit (*N* → ∞) and the case of 2 molecules (*N* = 2). If the reaction network does not include non-catalytic reactions (or, includes negligible non-catalytic reactions), we can use the formula (Equation 45a) to compare them. On the other hand, if the reaction network includes (non-negligible) non-catalytic reactions, we need to apply the formula, 2mDESM (Equation 72), with the SME (Equation 18) to obtain the concentrations in the case of 2 molecules. Although our theory has a presupposition referred to as *entire ergodicity*, the presupposition is intuitively verifiable if the system is specified, as in Section 2.4.2.4. We also demonstrated three examples for *non-autocatalyzation conversions of autocatalytic reaction networks* in Section 3.4. We consider this type of conversion to be generalized relatively easily such that our analytical framework can be applied to more general catalytic reaction networks including autocatalytic reactions.

One might think that the analysis presented in this paper can be straightforwardly extended to the case including autocatalytic reactions [in fact, the CME (Equation 7) and GFE (Equation 11) themselves hold even in the case including autocatalytic reactions]. However, if autocatalytic reactions are included (i.e., the case *R*_*ikk*_ > 0 is allowed), we cannot consider catalytic reactions and non-catalytic reactions to be completely separate. The reason is that, in the case including autocatalytic reactions, the absence of non-catalytic reactions generally implies winner-takes-all steady states. Generally, solving the CME (or GFE) of catalytic-noncatalytic mixed reactions systems is more advanced and a more difficult task than that of catalytic reactions only. The proposed strategy, i.e., non-autocatalyzation conversions, is one of our ideas to address the problem.

The formulas obtained in the present work are specific and satisfactorily simple. Therefore, our theory has the capabilities to be developed into a general theory for catalytic reaction networks. On the other hand, there exists a mathematical theory for a certain class of catalytic reaction networks that are “weakly reversible” and “deficiency zero” (Anderson et al., 2010). Our formulas (Equations 27 and 43) are consistent with the main theorems (theorem 4.1 and 4.2 in Anderson et al., 2010) in the above-mentioned mathematical theory. One of the advantages of our theory compared to the above mathematical theory is understandably the probability generating function (PGF) approach, because the PGF is a major analytical tool for physicists, chemists, and mathematical biologists. Therefore, our theory is easily verifiable, and one can design a computer algorithm to calculate our analytical formulas. We also showed the extensibility of our theory by using applications (in Section 3), especially because of the CStoWTAS (Equation 61), which was not suggested by the above mathematical theory.

Actual biochemical pathways in the cell involve thousands of chemical species, and their chemical properties vary. Our theoretical framework is general and extensible to such complex reaction networks, if they can be represented by CMEs such as Equation (7). As our current model consists of simple two-body catalytic reactions, it is difficult to point out examples in actual biological systems that correspond exactly to our model. Biochemical reactions in reality may involve a number of intermediates. There are also autocatalytic processes such as autophosphorylation, and replication of templates such as DNA, in which the catalyst or template species is also a substrate or a product. Our framework is applicable to many such cases involving network conversion, as shown for simple autocatalytic cases.

Nevertheless, the reaction kinetics of each enzyme is not always simple. Enzymes are complex macromolecules and their reaction cycles may depend on their conformational states. Therefore, the prediction of biological phenomena caused by small-number effects in real biochemical reactions, would entail further analytical challenges for catalytic reaction networks including arbitrary higher-order mixed reactions (rather than first- and second-order reactions only) or internal dynamics of the enzymes (as modeled and analyzed in Togashi and Casagrande, 2015) as important issues.

Throughout this work, our primary intention is to approach small-number issues in biological systems. One might wonder how general these small-number issues appear, and how important they are, in living cells. Recently, absolute quantification of various proteins and mRNAs in the cell has become possible, and the integration of experimental results (e.g., the construction of a database Milo et al., 2010) is also underway. Taniguchi et al. investigated the copy number distribution for more than a thousand protein species in bacteria (Taniguchi et al., 2010). Li et al. further discussed the relationship between the copy number and synthesis rate, and also the role, of proteins (Li et al., 2014). According to the result, some transcription factors, particularly activators, are rare, of the order of 0.1 to 10 molecules per genome equivalent. Although stochastic gene expression has been intensively discussed for years (McAdams and Arkin, 1997; Thattai and van Oudenaarden, 2001; Elowitz et al., 2002; Raj and van Oudenaarden, 2008; Shahrezaei and Swain, 2008), the discrete small-number nature of transcription factors has often been ignored; hence, the finding may urge us to reconsider the issue. Synthetic approaches are also becoming popular to confirm small-number effects. Ma et al. reported that an additional stable state in a genetic bistable toggle switch attributable to the small-number effect, which was predicted by stochastic simulations, was indeed observed in bacteria containing the genetically engineered switch (Ma et al., 2012). These results suggest that such rare proteins, of the order of one molecule per cell, are common and affect regulatory function in bacteria.

Although eukaryotic cells are much larger than bacteria, they have complex membrane structures and cytoskeletons inside, and the small-number issues can be particularly significant in compartments or bottlenecks (e.g., if we consider the volume of a synaptic vesicle represented by a sphere 40 nm in diameter, then 1 molecule corresponds to ca. 50 μmol/L). Rare proteins are also involved in physiologically important signaling pathways in eukaryotes. In the Wnt signaling pathway, for example, the concentration of axin is reported to be 20 pmol/L in Xenopus eggs (Lee et al., 2003) (though suggested to be higher in mammalian cells Tan et al., 2012). Another example is the MAP-kinase cascade, where proteins of the order of merely 10^2^ molecules (e.g., Ste5) exist in a yeast cell (Thomson et al., 2011). For scaffold proteins such as axin and Ste5, specifically, localization (locally high concentrations) of other chemical species around the scaffold may drastically change the reaction behavior, as spatial discreteness of the scaffolds becomes significant (Shnerb et al., 2000; Togashi and Kaneko, 2004). Further studies in which spatial structures (cf. reaction-diffusion equations) are considered are also expected to be important.

In the presented framework, we mainly focused on the steady-state solutions of GFE. Of course, temporal courses are biologically crucial in some cases. A well-studied example is oscillatory behavior in circadian clocks (Bell-Pedersen et al., 2005). In such oscillations, if a chemical factor is depleted down to a small number in a certain phase, then, the period can be susceptible to stochastic reactions involving the factor in that phase; on the other hand, sequestration of a factor may contribute to regular oscillations (Jolley et al., 2012). Again, the internal dynamics of enzymes can also be relevant in some systems. Although stochastic simulations are indeed powerful and many attempts are currently underway, further theoretical understanding as well as the experimental quantitative observation of rare factors would be required.

Note that a chemical “species” here can also be interpreted as a specific state of a molecule; e.g., we can consider proteins or genes, with and without modification, as separate species. Moreover, a similar interpretation is also applicable to ecology and ethology (Biancalani et al., 2014), if the laws governing the system are analogous to reactions. We remain hopeful that theoretical frameworks including ours will facilitate the exploration of small-number issues at equally higher levels of biological systems in future.

## Author contributions

MN and YT conceived the research. MN performed the analysis and simulations. Both authors discussed the results and wrote the paper.

## Funding

This work was supported by the Ministry of Education, Culture, Sports, Science, and Technology, Japan (KAKENHI 23115007 “Spying minority in biological phenomena”), and Japan Agency for Medical Research and Development (Platform for Dynamic Approaches to Living System).

### Conflict of interest statement

The authors declare that the research was conducted in the absence of any commercial or financial relationships that could be construed as a potential conflict of interest.
